# Supplementary material for: Analysis of Clinical Phenotypes through Machine Learning of First-Line H. pylori Treatment in Europe during the Period 2013–2022: Data from the European Registry on H. pylori Management (Hp-EuReg)
Source: Antibiotics (Basel). 2023 Sep 10;12(9):1427. doi: 10.3390/antibiotics12091427 (PMC10525558; doi:10.3390/antibiotics12091427)
Supplement: Supplementary file 1 [file antibiotics-12-01427-s001.zip › antibiotics-2568061-supplementary correct.pdf]

ONLINE SUPPLEMENTARY MATERIAL

Supplementary file S1. Hp-EuReg investigators

Manuel Pabón-Carrasco, Department of Gastroenterology, Hospital Universitario de Valme, Seville, SPAIN Acquired data, critically reviewed the manuscript draft, and approved the submitted manuscript.

Alma Keco-Huerga, Department of Gastroenterology, Hospital Universitario de Valme, Seville, SPAIN Acquired data, critically reviewed the manuscript draft, and approved the submitted manuscript.

Alfredo J. Lucendo, Centro de Investigación Biomédica en Red en Enfermedades Digestivas y Hepáticas (CIBERehd); Department of Gastroenterology, Instituto de Investigación Sanitaria Princesa (IIS-IP); Department of Gastroenterology, Hospital General de Tomelloso, Tomelloso, SPAIN Acquired data, critically reviewed the manuscript draft, and approved the submitted manuscript.

Maja Denkovski, Department of Gastroenterology, Interni Oddelek, Diagnostic Centre, Bled, SLOVENIA Acquired data, critically reviewed the manuscript draft, and approved the submitted manuscript.

Natasa Brglez Jurecic, Department of Gastroenterology, Interni Oddelek, Diagnostic Centre, Bled, SLOVENIA Acquired data, critically reviewed the manuscript draft, and approved the submitted manuscript.

Ludmila Vologzanina, Gastrocentr, Perm, RUSSIA Acquired data, critically reviewed the manuscript draft, and approved the submitted manuscript.

Luis Rodrigo, Department of Gastroenterology, University of Oviedo, Oviedo, SPAIN Acquired data, critically reviewed the manuscript draft, and approved the submitted manuscript.

Renate Bumane, Department of Gastroenterology, Digestive Diseases Centre Gastro, Institute of Clinical and Preventive Medicine and Faculty of Medicine, University of Latvia, Riga, LATVIA Acquired data, critically reviewed the manuscript draft, and approved the submitted manuscript.

Rustam Abdulkhakov, Department of Hospital Medicine, Kazan State Medical University, Kazan, RUSSIA Acquired data, critically reviewed the manuscript draft, and approved the submitted manuscript.

Sayar Abdulkhakov, Department of Hospital Medicine, Kazan State Medical University, Kazan, RUSSIA Acquired data, critically reviewed the manuscript draft, and approved the submitted manuscript.

Thomas J. Butler, Department of Clinical Medicine, Trinity College Dublin, Department of Gastroenterology, Tallaght University Hospital, Dublin, IRELAND Acquired data, critically reviewed the manuscript draft, and approved the submitted manuscript.

Rebecca FitzGerald, School of Medicine, Trinity College Dublin, Dublin, IRELAND Acquired data, critically reviewed the manuscript draft, and approved the submitted manuscript.

Deirdre McNamara, School of Medicine, Trinity College Dublin, Dublin, IRELAND Acquired data, critically reviewed the manuscript draft, and approved the submitted manuscript

Ana Garre, Hospital Universitario de La Princesa, Instituto de Investigación Sanitaria Princesa (IIS-Princesa), Universidad Autónoma de Madrid (UAM), Centro de Investigación Biomédica en Red de Enfermedades Hepáticas y Digestivas (CIBERehd), Madrid, SPAIN Acquired data, critically reviewed the manuscript draft, and approved the submitted manuscript

Galyna Fadieienko, Departments the Division for the Study of the Digestive diseases and its Comorbidity with Noncommunicable Diseases, Government Institution L.T. Malaya Therapy National Institute of NAMS of Ukraine, Kharkiv, UKRAINE Acquired data, critically reviewed the manuscript draft, and approved the submitted manuscript

Jose M. Huguet, Patología Digestiva, Hospital General Universitario de Valencia, Valencia, SPAIN Acquired data, critically reviewed the manuscript draft, and approved the submitted manuscript.

Oleg Zaytsev, Gastroenterology Unit, First Clinical Medical Center Kovrov, Kovrov, RUSSIA Acquired data, critically reviewed the manuscript draft, and approved the submitted manuscript.

Aiman Silkanovna Sarsenbaeva, Department of Gastroenterology, Chelyabinsk Regional Clinical Hospital, Chelyabinsk, RUSSIA Acquired data, critically reviewed the manuscript draft, and approved the submitted manuscript.

L. Fernández-Salazar, Gastroenterology Department, Hospital Clínico Universitario de Valladolid (SACYL); Medicine Department, School of Medicine, Universidad de Valladolid, Valladolid, SPAIN Acquired data, critically reviewed the manuscript draft, and approved the submitted manuscript.

B. Velayos, Gastroenterology Department, Hospital Clínico Universitario Valladolid, Valladolid, SPAIN Acquired data, critically reviewed the manuscript draft, and approved the submitted manuscript.

Noelia Alcaide, Gastroenterology Department, Hospital Clínico Universitario Valladolid, Valladolid, SPAIN Acquired data, critically reviewed the manuscript draft, and approved the submitted manuscript.

Ilchishina Tatiana, Gastroenterology Unit, SM-clinic, Saint-Petersburg, RUSSIA Acquired data, critically reviewed the manuscript draft, and approved the submitted manuscript.

Igor Bakulin, North-western State Medical University, Peter The Great Clinical Hospital, Saint-Petersburg, RUSSIA Acquired data, critically reviewed the manuscript draft, and approved the submitted manuscript.

Marco Romano, Gastroenterology and Endoscopy Unit, Dipartimento di Medicina di Precisione, Università Vanvitelli, Napoli, ITALY Acquired data, critically reviewed the manuscript draft, and approved the submitted manuscript.

Antonietta G. Gravina, Hepatogastroenterology Unit, Università Vanvitelli, Napoli, ITALY Acquired data, critically reviewed the manuscript draft, and approved the submitted manuscript.

Monica Perona, Gastroenterology Department, Hospital Quironsalud Marbella, Marbella, SPAIN Acquired data, critically reviewed the manuscript draft, and approved the submitted manuscript.

Miguel Areia, Faculty of Medicine of the University of Porto (FMUP), Center for Health Technology and Services Research (CINTESIS); Department of

Gastroenterology, Portuguese Oncology Institute Coimbra, Coimbra, PORTUGAL Acquired data, critically reviewed the manuscript draft, and approved the submitted manuscript.

Pavel Bogomolov, Universal Clinic Private Medical Center, Moscow, RUSSIA Acquired data, critically reviewed the manuscript draft, and approved the submitted manuscript.

Sergey Alekseenko, Far Eastern State Medical University, Khabarovsk, RUSSIA Acquired data, critically reviewed the manuscript draft, and approved the submitted manuscript.

Jesús Barrio, Gastroenterology Department, Hospital Universitario Río Hortega, Gerencia Regional de Salud de Castilla y León (SACYL), Valladolid, SPAIN Acquired data, critically reviewed the manuscript draft, and approved the submitted manuscript.

Óscar Nuñez, Digestive Service, Hospital Universitario Sanitas La Moraleja, Madrid, SPAIN Acquired data, critically reviewed the manuscript draft, and approved the submitted manuscript.

Javier Tejedor-Tejada, Department of Gastroenterology, Hospital Universitario de Cabueñes, Gijón, Asturias, SPAIN Acquired data, critically reviewed the manuscript draft, and approved the submitted manuscript.

Pilar Mata-Romero, Servicio de Aparato Digestivo, Hospital Universitario de Cáceres, Cáceres, SPAIN Acquired data, critically reviewed the manuscript draft, and approved the submitted manuscript.

D. Martin-Holgado, Servicio de Aparato Digestivo, Hospital Universitario de Cáceres, Cáceres, SPAIN Acquired data, critically reviewed the manuscript draft, and approved the submitted manuscript.

Blas José Gómez Rodríguez, Gastroenterology Department, Hospital Universitario Virgen de la Macarena, Seville, SPAIN Acquired data, critically reviewed the manuscript draft, and approved the submitted manuscript.

Diego Ledro-Cano, Gastroenterology Department, Hospital Universitario Virgen de la Macarena, Seville, SPAIN Acquired data, critically reviewed the manuscript draft, and approved the submitted manuscript.

Inmaculada Ortiz-Polo, Gastroenterology Department, Hospital Universitario y Politécnico la Fe, Valencia, SPAIN Acquired data, critically reviewed the manuscript draft, and approved the submitted manuscript.

Sotirios D. Georgopoulos, Gastroenterology Department, Athens Medical, P. Faliron Hospital, Athens, GREECE Acquired data, critically reviewed the manuscript draft, and approved the submitted manuscript.

György Miklós Buzás, Gastroenterology, Ferencváros Health Center, Budapest, HUNGARY Acquired data, critically reviewed the manuscript draft, and approved the submitted manuscript.

Manuel Domínguez Cajal, Gastroenterology and Hepatology Unit, Hospital Universitario San Jorge, Huesca, SPAIN Acquired data, critically reviewed the manuscript draft, and approved the submitted manuscript.

Galina Tarasova, Department of Gastroenterology, Rostov State Medical University, Rostov-on-Don, RUSSIA Acquired data, critically reviewed the manuscript draft, and approved the submitted manuscript.

Cem Simsek, Department of Gastroenterology, Hacettepe University, Mehmet Akif Inan, Health Sciences University, Medical Center, HC International Clinic, Ankara, TURKEY Acquired data, critically reviewed the manuscript draft, and approved the submitted manuscript.

Rinaldo Pellicano, Unit of Gastroenterology, Molinette-SGAS Hospital, Turin, ITALY Acquired data, critically reviewed the manuscript draft, and approved the submitted manuscript.

Pedro Almela, Digestive Service, Hospital General Universitario de Castellón, Ciencias de la Salud, Universidad CEU Cardenal Herrera, Castellón, SPAIN Acquired data, critically reviewed the manuscript draft, and approved the submitted manuscript.

Gema Ladrón Villanueva, Digestive Service, Hospital General Universitario de Castellón, Castellón, SPAIN Acquired data, critically reviewed the manuscript draft, and approved the submitted manuscript.

Leticia Gimeno Pitarch, Digestive Service, Hospital General Universitario de Castellón, Castellón, SPAIN Acquired data, critically reviewed the manuscript draft, and approved the submitted manuscript.

Antonio Mestrovic, Department of Gastroenterology, University Hospital of Split, Split, CROATIA Acquired data, critically reviewed the manuscript draft, and approved the submitted manuscript.

Francesco Franceschi, Emergency Medicine, Fondazione Policlinico Universitario "A. Gemelli" IRCCS, Università Cattolica del Sacro Cuore, Rome, ITALY Acquired data, critically reviewed the manuscript draft, and approved the submitted manuscript.

Boris D Starostin, Saint-Petersburg State Budgetary Institution Healthcare City Policlinic 38, Saint-Petersburg, RUSSIA Acquired data, critically reviewed the manuscript draft, and approved the submitted manuscript.

N.N. Dekhnich, Department of Faculty Therapy, Smolensk State Medical University, Smolensk, RUSSIA Acquired data, critically reviewed the manuscript draft, and approved the submitted manuscript.

Judith Gomez-Camarero, Gastroenterology Department, Hospital Universitario de Burgos, Burgos, SPAIN Acquired data, critically reviewed the manuscript draft, and approved the submitted manuscript.

Alla Kononova, Tver State Madical University, Tver, RUSSIA Acquired data, critically reviewed the manuscript draft, and approved the submitted manuscript.

Marinko Marušić, Department of gastroenterology, University Hospital Sveti Duh, School of Medicine, University J. J. Strossmayer Osijek, Faculty of Health Studies, University of Rijeka,Rijeka, CROATIA Acquired data, critically reviewed the manuscript draft, and approved the submitted manuscript.

Montserrat Planella, Service of Gastroenterology, Hospital Universitari Arnau de Vilanova; Institut de Recerca Biomèdica de Lleida (IRBLL), Lleida, SPAIN Acquired data, critically reviewed the manuscript draft, and approved the submitted manuscript.

Consuelo Ramirez, Service of Gastroenterology, Hospital Universitari Arnau de Vilanova; Institut de Recerca Biomèdica de Lleida (IRBLL), Lleida, SPAIN Acquired data, critically reviewed the manuscript draft, and approved the submitted manuscript.

Eduardo Iyo, Digestive Service, Hospital Comarcal de Inca, Inca, SPAIN Acquired data, critically reviewed the manuscript draft, and approved the submitted manuscript.

Miguel Fernández-Bermejo, Digestive Service, Clínica San Francisco, Cáceres, SPAIN Acquired data, critically reviewed the manuscript draft, and approved the submitted manuscript.

Rafael Ruiz-Zorrilla Lopez, Gastroenterology & Hepatology Department, Sierrallana Hospital, Torrelavega, SPAIN Acquired data, critically reviewed the manuscript draft, and approved the submitted manuscript.

Pablo Miles Wolfe García, Gastroenterology & Hepatology Department, Sierrallana Hospital, Torrelavega, SPAIN Acquired data, critically reviewed the manuscript draft, and approved the submitted manuscript.

Rosa Rosania, Department of Gastroenterology, Hepatology and Infectious Diseases, Otto von Guericke University Hospital, Magdeburg, GERMANY Acquired data, critically reviewed the manuscript draft, and approved the submitted manuscript.

Joana Alves Silva, Department of Gastroenterology, Centro Hospitalar Universitário do Porto, Porto, PORTUGAL Acquired data, critically reviewed the manuscript draft, and approved the submitted manuscript.

Ludmila Morkovkina, Chuvash Republic Hospital, Cheboksary, RUSSIA Acquired data, critically reviewed the manuscript draft, and approved the submitted manuscript.

Javier Alcedo, Department of Gastroenterology, Hospital Universitario Miguel Servet, Zaragoza, SPAIN Acquired data, critically reviewed the manuscript draft, and approved the submitted manuscript.

Alfredo Di Leo, Section of Gastroenterology, Department of Precision and Regenerative Medicine and Ionian Area, University of Bari, Bari, ITALY Acquired data, critically reviewed the manuscript draft, and approved the submitted manuscript.

Giuseppe Losurdo, Section of Gastroenterology, Department of Precision and Regenerative Medicine and Ionian Area, University of Bari, Bari, ITALY Acquired data, critically reviewed the manuscript draft, and approved the submitted manuscript.

Dan L. Dumitrascu, Second Department of Internal Medicine, Iuliu Hatieganu University of Medicine and Pharmacy Cluj-Napoca, Cluj-Napoca, ROMANIA Acquired data, critically reviewed the manuscript draft, and approved the submitted manuscript.

Fernando Bermejo, Digestive Service, Hospital Universitario de Fuenlabrada, Instituto de Investigación Sanitaria La Paz (idiPAZ), Madrid, SPAIN Acquired data, critically reviewed the manuscript draft, and approved the submitted manuscript.

Ian L. P. Beales, Norwich Medical School, University of East Anglia, Norwich, United Kingdom Acquired data, critically reviewed the manuscript draft, and approved the submitted manuscript.

Itxaso Jiménez, Gastroenterology Department, Hospital Universitario de Galdakao-Usansolo, Galdakao, SPAIN Acquired data, critically reviewed the manuscript draft, and approved the submitted manuscript.

Georges Kamtoh, Hepatic Medical, Private Medical Center, Krakow, POLAND Acquired data, critically reviewed the manuscript draft, and approved the submitted manuscript.

Ramón Pajares Villarroya, Department of Gastroenterology, Hospital Universitario Infanta Sofía, San Sebastián de los Reyes, SPAIN Acquired data, critically reviewed the manuscript draft, and approved the submitted manuscript.

Paola Chaudarcas, Department of Gastroenterology, Hospital Universitario Infanta Sofía, San Sebastián de los Reyes, SPAIN Acquired data, critically reviewed the manuscript draft, and approved the submitted manuscript.

T. Angueira, Department of Gastroenterology, Hospital General de Tomelloso, Tomelloso, SPAIN Acquired data, critically reviewed the manuscript draft, and approved the submitted manuscript.

Victor A. Kamburov, Department of Gastroenterology, BalkanMed Medical Center, Sofia, BULGARIA Acquired data, critically reviewed the manuscript draft, and approved the submitted manuscript.

Natalia Baryshnikova, Internal disease department of stomatology faculty, Pavlov First Saint Petersburg State Medical University; Molecular microbiology

department, Institute of Experimental Medicine, Saint-Petersburg, RUSSIA Acquired data, critically reviewed the manuscript draft, and approved the submitted manuscript.

Antonio Moreno Loro, Gastroenterology Department, Hospital Universitario Virgen del Rocío, Seville, SPAIN Acquired data, critically reviewed the manuscript draft, and approved the submitted manuscript.

L. de la Peña, Gastroenterology Department, Viladecans Hospital, Viladecans, SPAIN Acquired data, critically reviewed the manuscript draft, and approved the submitted manuscript.

Sabir Sagdati, Gastroenterologija, Endoskopija, Gastromedica GM, Novi Pazar, SERBIA Acquired data, critically reviewed the manuscript draft, and approved the submitted manuscript.

Natalia V. Bakanova, Medical Center Mediceya, Izhevsk, RUSSIA Acquired data, critically reviewed the manuscript draft, and approved the submitted manuscript.

Ana Campillo, Hospital Reina Sofía, Tudela, SPAIN Acquired data, critically reviewed the manuscript draft, and approved the submitted manuscript.

Matteo Ghisa, Gastroenterology Unit, Department of Surgery, Oncology and Gastroenterology, University of Padua, Gastroenterology Unit, Department of Oncological Gastrointestinal Surgery, S. Maria del Prato Hospital, Padua, ITALY Acquired data, critically reviewed the manuscript draft, and approved the submitted manuscript.

Fabio Farinati, Gastroenterology Unit, Department of Surgery, Oncology and Gastroenterology, University of Padua, Padua, ITALY Acquired data, critically reviewed the manuscript draft, and approved the submitted manuscript.

Jitka Vaculova, Department of Gastroenterology and Internal Medicine, Department of Surgery, University Hospital Brno, Faculty of Medicine, Masaryk University, Brno, CZECH REPUBLIC Acquired data, critically reviewed the manuscript draft, and approved the submitted manuscript.

M. Soledad Marcos, Gastroenterology Department, Hospital 12 de Octubre, Madrid, SPAIN Acquired data, critically reviewed the manuscript draft, and approved the submitted manuscript.

Debora Compare, Gastroenterology Unit, Department of Clinical Medicine and Surgery, University Federico II of Naples, Naples, ITALY Acquired data, critically reviewed the manuscript draft, and approved the submitted manuscript.

Piotr Eder, Department of Gastroenterology, Dietetics and Internal Medicine, Poznań University of Medical Sciences; Department of Gastroenterology, H. Święcicki University Hospital, Poznan, POLAND Acquired data, critically reviewed the manuscript draft, and approved the submitted manuscript.

Dmitrii Andreev, A.I. Yevdokimov Moscow State University of Medicine and Dentistry, Moscow, RUSSIA Acquired data, critically reviewed the manuscript draft, and approved the submitted manuscript.

Igor Maev, A.I. Yevdokimov Moscow State University of Medicine and Dentistry, Moscow, RUSSIA Acquired data, critically reviewed the manuscript draft, and approved the submitted manuscript.

Jurij Bednarik, Gastroenterology Department, Klinika Doktor 24, Ljubljana, SLOVENIA Acquired data, critically reviewed the manuscript draft, and approved the submitted manuscript.

Sabina Hrubá, University Hospital Pilsen, Pilsen, CZECH REPUBLIC Acquired data, critically reviewed the manuscript draft, and approved the submitted manuscript.

Jürg Knuchel, Division of Gastroenterology and Hepatology, Medical University Department, Kantonsspital Aarau, Aarau, SWITZERLAND Acquired data, critically reviewed the manuscript draft, and approved the submitted manuscript.

Thomas Kuntzen, Division of Gastroenterology and Hepatology, Medical University Department, Kantonsspital Aarau, Aarau, SWITZERLAND Acquired data, critically reviewed the manuscript draft, and approved the submitted manuscript.

Eva Hefti, Division of Gastroenterology and Hepatology, Medical University Department, Kantonsspital Aarau, Aarau, SWITZERLAND Acquired data, critically reviewed the manuscript draft, and approved the submitted manuscript.

Jan Kral, Medic Kral s.r.o., Prague, CZECH REPUBLIC Acquired data, critically reviewed the manuscript draft, and approved the submitted manuscript.

Barbara Gomez, Gastroenterology Unit, Hospital de Mataró, Barcelona, SPAIN Acquired data, critically reviewed the manuscript draft, and approved the submitted manuscript.

Natalia Valerievna, I.I. Mechnikov North-Western State Medical University, Saint Petersburg, RUSSIA Acquired data, critically reviewed the manuscript draft, and approved the submitted manuscript.

Liya Nikolaevna Belousova, I.I. Mechnikov North-Western State Medical University, Saint Petersburg, RUSSIA Acquired data, critically reviewed the manuscript draft, and approved the submitted manuscript.

Christos Liatsos, Gastroenterology Department, 401 Military Hospital of Athens, Athens, GREECE Acquired data, critically reviewed the manuscript draft, and approved the submitted manuscript.

Piotr Szredzki, John Paul II City Hospital, Rzeszów, POLAND Acquired data, critically reviewed the manuscript draft, and approved the submitted manuscript.

Wojciech Jakub Siwiaszczyk, John Paul II City Hospital, Rzeszów, POLAND Acquired data, critically reviewed the manuscript draft, and approved the submitted manuscript.

Ivan Nagorni, Clinical Centre of Niš, Niš, SERBIA Acquired data, critically reviewed the manuscript draft, and approved the submitted manuscript.

Alisan Kahraman, Gastroenterology and Hepatology Department, Max Grundig Clinic, Bühl, GERMANY Acquired data, critically reviewed the manuscript draft, and approved the submitted manuscript.

Nayden Marinov Kandilarov, Aleksandrovska University Hospital, Sofia, BULGARIA Acquired data, critically reviewed the manuscript draft, and approved the submitted manuscript.

Isabel Pérez-Martínez, Department of Gastroenterology, Hospital Universitario Central de Asturias; Instituto de Investigación Sanitaria del Principado de Asturias (ISPA), Oviedo, SPAIN Acquired data, critically reviewed the manuscript draft, and approved the submitted manuscript.

Ekaterina Yuryevna Plotnikova, Kemerovo State Medical Academy, Kemerovo, RUSSIA Acquired data, critically reviewed the manuscript draft, and approved the submitted manuscript.

Virginia Flores, Gastroenterology Department, Hospital General Universitario Gregorio Marañón, Madrid, SPAIN Acquired data, critically reviewed the manuscript draft, and approved the submitted manuscript.

Riccardo Vasapolli, Medical Department 2, University Hospital LMU Munich, Munich, GERMANY Acquired data, critically reviewed the manuscript draft, and approved the submitted manuscript.

F. Rancel Medina, Digestive Service, Complejo Asistencial Universitario de Palencia, Palencia, SPAIN Acquired data, critically reviewed the manuscript draft, and approved the submitted manuscript.

Nikola Panic, Medical Faculty University of Belgrade, University Medical Centre Dr Dragiša Mišović, Belgrade, SERBIA Acquired data, critically reviewed the manuscript draft, and approved the submitted manuscript.

Mila Kovacheva-Slavova, Department of Gastroenterology, Clinic of Gastroenterology, University Hospital 'Tsaritsa Yoanna-ISUL', Medical University of Sofia, Sofia, BULGARIA Acquired data, critically reviewed the manuscript draft, and approved the submitted manuscript.

Joan Llach, Gastroenterology Department, Hospital Clínic de Barcelona, Barcelona, SPAIN Acquired data, critically reviewed the manuscript draft, and approved the submitted manuscript.

Regina I. Khlynova, Department of faculty therapy and geriatrics, Ural State Medical University, Ekaterinburg, RUSSIA Acquired data, critically reviewed the manuscript draft, and approved the submitted manuscript.

Olga Kolokolnikova, Medsi Clinical Hospital, Moscow, RUSSIA Acquired data, critically reviewed the manuscript draft, and approved the submitted manuscript.

Marko Nikolić, University Hospital Centar Sestre Milosrdnice, Zagreb, CROATIA Acquired data, critically reviewed the manuscript draft, and approved the submitted manuscript.

Antonio Cuadrado, Department of Gastroenterology and Hepatology, Marqués de Valdecilla University Hospital, Clinical and Translational Research in Digestive Diseases, Valdecilla Research Institute (IDIVAL), Santander, SPAIN Acquired data, critically reviewed the manuscript draft, and approved the submitted manuscript.

Petra Čavajdová, University Hospital Hradec Kralove, Hradec Kralove, CZECH REPUBLIC Acquired data, critically reviewed the manuscript draft, and approved the submitted manuscript.

Melanija Razov Radas, Department of Gastroenterology and Hepatology, General Hospital Zadar, Health Studies, University of Zadar, Zadar, CROATIA Acquired data, critically reviewed the manuscript draft, and approved the submitted manuscript.

Ana Beatriz Pozo Blanco, Department of Gastroenterology, Hospital Arnau Vilanova-Lliria, Valencia, SPAIN Acquired data, critically reviewed the manuscript draft, and approved the submitted manuscript.

Daniele A. Riva, Department of Gastroenterology, Gastrocentro Plus, Lugano, SWITZERLAND Acquired data, critically reviewed the manuscript draft, and approved the submitted manuscript.

Ivana Milicevic, University Clinic for Gastroenterohepatology, Faculty of Medicine, Ss. Cyril and Methodius University in Skopje, Skopje, REPUBLIC OF NORTH MACEDONIA Acquired data, critically reviewed the manuscript draft, and approved the submitted manuscript.

Dafina Nikolova, University Clinic of Gastroenterohepatology, Faculty of Medicine, University "Ss. Cyril and Methodius", Skopje, REPUBLIC OF NORTH MACEDONIA Acquired data, critically reviewed the manuscript draft, and approved the submitted manuscript.

Manuel Jiménez-Moreno, Gastroenterology Department, Hospital Santos Reyes, Aranda de Duero, SPAIN Acquired data, critically reviewed the manuscript draft, and approved the submitted manuscript.

Stergios N. Kouvaras, Refferal Endoscopy Unit, Private Endoscopy Unit, Halkida, GREECE Acquired data, critically reviewed the manuscript draft, and approved the submitted manuscript.

Katarina Jankovic, Clinic for Gastroenterohepatology, University Clinical Centre of Serbia, Belgrade, SERBIA Acquired data, critically reviewed the manuscript draft, and approved the submitted manuscript.

Anna Pakhomova, Saratov State Clinical Hospital, Saratov, RUSSIA Acquired data, critically reviewed the manuscript draft, and approved the submitted manuscript.

Diego Burgos-Santamaría, Department of Gastroenterology and Hepatology, Hospital Universitario Ramón y Cajal, Madrid, SPAIN Acquired data, critically reviewed the manuscript draft, and approved the submitted manuscript.

Jose Xavier Segarra Ortega, Department of Gastroenterology, Hospital Universitario de Salamanca, Grupo GISAD, Instituto de Investigación Biomédica de Salamanca, Salamanca, SPAIN Acquired data, critically reviewed the manuscript draft, and approved the submitted manuscript.

Jesus M. Gonzalez-Santiago, Department of Gastroenterology, Complejo Asistencial Universitario de Salamanca, Instituto de Investigación Biomédica de Salamanca (IBSAL), Centro de Investigación Biomédica en Red de Enfermedades Hepáticas y Digestivas (CIBERehd), Salamanca, SPAIN Acquired data, critically reviewed the manuscript draft, and approved the submitted manuscript.

Marija Branković, Department of Internal Medicine, University Hospital Medical Center Bežanijska kosa, Faculty of Medicine, University of Belgrade, Belgrade, SERBIA Acquired data, critically reviewed the manuscript draft, and approved the submitted manuscript.

W. Soub Defeu, Department of Gastroenterology, CHU Charleroi, Charleroi, BELGIUM Acquired data, critically reviewed the manuscript draft, and approved the submitted manuscript.

Kateryna Priadko, Hepato-Gastroenterology & Digestive Oncology Unit, University Hospital of Nantes, Nantes, FRANCE Acquired data, critically reviewed the manuscript draft, and approved the submitted manuscript.

A. Vasura, Department of Gastroenterology, Hepatology and Pancreatology, Internal and cardiologic Clinic, University hospital of Ostrava, Ostrava, CZECH REPUBLIC Acquired data, critically reviewed the manuscript draft, and approved the submitted manuscript.

Mirjana Kalauz, Endoscopy Unit, Division of Gastroenterology, Department of Internal Medicine, Clinical Hospital Center Zagreb, Internal Medicine, School of Medicine, University of Zagreb, Zagreb, CROATIA Acquired data, critically reviewed the manuscript draft, and approved the submitted manuscript.

Michal Kukla, Department of Internal Medicine and Geriatrics, Faculty of Medicine, Jagiellonian University Medical College, Department of Endoscopy, University Hospital, Krakow, POLAND Acquired data, critically reviewed the manuscript draft, and approved the submitted manuscript.

Dušan Kekić, Bacteriology Unit, Institute for microbiology and immunology, Medical Faculty, University of Belgrade, Belgrade, SERBIA Acquired data, critically reviewed the manuscript draft, and approved the submitted manuscript.

Goran Hauser, Internal medicine, Faculty of Medicine University of Rijeka, Gastroenterology Unit, Clinical Hospital Centre Rijeka, Rijeka, CROATIA Acquired data, critically reviewed the manuscript draft, and approved the submitted manuscript.

Idaira Fernández Cabrera, Department of Gastroenterology, Hospital Universitario de Gran Canaria Doctor Negrín, Las Palmas de Gran Canaria,

SPAIN Acquired data, critically reviewed the manuscript draft, and approved the submitted manuscript.

Carlos Rodríguez Pérez, Department of Gastroenterology, Hospital Universitario de Gran Canaria Doctor Negrín, Las Palmas de Gran Canaria, SPAIN Acquired data, critically reviewed the manuscript draft, and approved the submitted manuscript.

Rachel Gingold-Belfer, Sackler Faculty of Medicine, Tel-Aviv University, Gastroenterology Division, Rabin Medical Center – Beilinson Hospital, Petach-Tikva, ISRAEL Acquired data, critically reviewed the manuscript draft, and approved the submitted manuscript.

Petra Koňaříková, Tomas Bata Regional Hospital, Zlin, CZECH REPUBLIC Acquired data, critically reviewed the manuscript draft, and approved the submitted manuscript.

Table S1. Summary description of patients and variables in each cluster, corresponding to the year 2013.

	1 (n=2,011)	2 (n=224)	3 (n=1,004)	Overall p-value	N
**Gastrointestinal symptoms**				<0.001	3,239
Absence (none)	1,706 (84.8%)	187 (83.5%)	693 (69.0%)		
Other symptoms[1]	305 (15.2%)	37 (16.5%)	311 (31.0%)		
**Compliance**				<0.001	3,239
No (< 90% drug intake)	104 (5.17%)	2 (0.89%)	8 (0.80%)		
Yes (> 90% drug intake)	1,907 (94.8%)	222 (99.1%)	996 (99.2%)		
**Duration (days)**				0.000	3,239
7	6 (0.30%)	21 (9.38%)	778 (77.5%)		
10	1,544 (76.8%)	185 (82.6%)	171 (17.0%)		
14	461 (22.9%)	18 (8.04%)	55 (5.48%)		
**Dose of PPI (mg OE)[2]**				<0.001	3,239
Low	1,025 (51.0%)	157 (70.1%)	833 (83.0%)		
Standard	410 (20.4%)	41 (18.3%)	82 (8.17%)		
High	576 (28.6%)	26 (11.6%)	89 (8.86%)		
**Country**					3,239
Belgium	20 (0.99%)	0 (0.00%)	2 (0.20%)		
Bulgaria	0 (0.00%)	0 (0.00%)	1 (0.10%)		
Croatia	74 (3.68%)	0 (0.00%)	2 (0.20%)		
Denmark	1 (0.05%)	0 (0.00%)	6 (1.59%)		
Finland	0 (0.00%)	0 (0.00%)	3 (0.30%)		
France	46 (2.29%)	0 (0.00%)	1 (0.10%)		
Germany	0 (0.00%)	0 (0.00%)	2 (0.20%)		
Greece	129 (6.41%)	0 (0.00%)	3 (0.30%)		
Hungary	1 (0.05%)	0 (0.00%)	7 (1.69%)		
Ireland	4 (0.20%)	0 (0.00%)	36 (3.59%)		
Israel	1 (0.05%)	0 (0.00%)	0 (0.00%)		
Italy	194 (9.65%)	0 (0.00%)	14 (1.39%)		
Latvia	1 (0.05%)	0 (0.00%)	107 (10.7%)		
Lithuania	1 (0.05%)	0 (0.00%)	36 (3.59%)		
Norway	3 (0.15%)	0 (0.00%)	273 (27.2%)		
Poland	0 (0.00%)	0 (0.00%)	11 (1.10%)		
Portugal	27 (1.34%)	0 (0.00%)	0 (0.00%)		
Romania	8 (0.40%)	0 (0.00%)	1 (0.10%)		
Russia	0 (0.00%)	223 (99.6%)	0 (0.00%)		
Slovenia	17 (0.85%)	0 (0.00%)	305 (30.4%)		
Spain	1,457 (72.5%)	1 (0.45%)	53 (5.28%)		
Switzerland	0 (0.00%)	0 (0.00%)	19 (1.89%)		
The Netherlands	0 (0.00%)	0 (0.00%)	13 (1.29%)		
Turkey	3 (0.15%)	0 (0.00%)	67 (6.67%)		
Ukraine	24 (1.19%)	0 (0.00%)	13 (1.29%)		

United Kingdom	0 (0.00%)	0 (0.00%)	9 (0.90%)	
**Most frequent 1st line treatments**				3,239
Triple-CA/M	589 (29.3%)	109 (48.7%)	753 (75.0%)	
Seq-CAT-CAM	558 (27.7%)	2 (0.89%)	1 (0.10%)	
Conco-CAT CAM	717 (35.7%)	3 (1.34%)	13 (1.29%)	
BsQuad-MTcB (including single capsule)	65 (3.23%)	3 (1.34%)	1 (0.10%)	
Quadruple-CAB	0 (0.00%)	63 (28.1%)	0 (0.00%)	
Other[3]	82 (4.08%)	44 (19.6%)	236 (23.5%)	

A: amoxicillin; B: bismuth salts; C: clarithromycin; Conco: concomitant; M: metronidazole; N: total number of cases in year evaluated; n: number of cases in each cluster; OE: omeprazole equivalent; PPI: proton pump inhibitor; Seq, sequential; T: tinidazole; Tc: tetracycline hydrochloride; MTcB was prescribed either in the classical form or as three-in-one single capsule, marketed as Pylera®. [1]Other gastrointestinal symptoms (excluding the most frequent ones such as dyspepsia or heartburn) included nausea, diarrhoea and weight loss. [2]Low dose PPI: 4.5–27 mg omeprazole equivalents, two times per day (i.e., 20 mg omeprazole equivalents, two times per day); standard dose PPI: 32–40 mg omeprazole equivalents, two times per day (i.e., 40 mg omeprazole equivalents, two times per day); high dose PPI: 54–128 mg omeprazole equivalents, two times per day (i.e., 80 mg omeprazole equivalents, two times per day).[3]Other treatments encompassed less than 10% of the remaining prescribed regimens. statistical significance was set at p-value <0.05**.**

Table S2. Summary description of patients and variables in each cluster, corresponding to the year 2014.

	1 (n=435)	2 (n=2,644)	3 (n=1,213)	Overall p-value	N
**Gastrointestinal symptoms**				<0.001	4,292
Absence (none)	404 (92.9%)	2,252 (85.2%)	969 (79.9%)		
Other symptoms[1]	31 (7.13%)	392 (14.8%)	244 (20.1%)		
**Compliance**				0.185	4,292
No (< 90% drug intake)	6 (1.38%)	38 (1.44%)	9 (0.74%)		
Yes (> 90% drug intake)	429 (98.6%)	2,606 (98.6%)	1,204 (99.3%)		
**Duration (days)**				0.000	4,292
7	1 (0.23%)	23 (0.87%)	977 (80.5%)		
10	433 (99.5%)	1,929 (73.0%)	164 (13.5%)		
14	1 (0.23%)	692 (26.2%)	72 (5.94%)		
**Dose of PPI (mg OE)[2]**				<0.001	4,292
Low	150 (34.5%)	1,059 (40.1%)	1,089 (89.8%)		
Standard	26 (5.98%)	911 (34.5%)	59 (4.86%)		
High	259 (59.5%)	674 (25.5%)	65 (5.36%)		
**Country**					4,292
Belgium	0 (0.00%)	42 (1.59%)	1 (0.08%)		
Croatia	9 (2.07%)	0 (0.00%)	0 (0.00%)		
Czech Republic	0 (0.00%)	1 (0.04%)	0 (0.00%)		
Denmark	0 (0.00%)	0 (0.00%)	3 (0.25%)		
Finland	0 (0.00%)	0 (0.00%)	2 (0.16%)		
France	42 (9.66%)	0 (0.00%)	0 (0.00%)		
Germany	0 (0.00%)	0 (0.00%)	3 (0.25%)		
Greece	32 (7.36%)	102 (3.86%)	4 (0.33%)		
Hungary	0 (0.00%)	8 (0.30%)	30 (2.47%)		
Ireland	44 (10.1%)	12 (0.45%)	49 (4.04%)		
Italy	280 (64.4%)	0 (0.00%)	9 (0.74%)		
Latvia	0 (0.00%)	33 (1.25%)	127 (10.5%)		
Lithuania	0 (0.00%)	0 (0.00%)	37 (3.05%)		
Norway	0 (0.00%)	16 (0.61%)	240 (19.8%)		
Poland	0 (0.00%)	1 (0.04%)	0 (0.00%)		
Portugal	10 (2.30%)	0 (0.00%)	0 (0.00%)		
Romania	0 (0.00%)	0 (0.00%)	7 (0.58%)		
Russia	1 (0.23%)	342 (12.9%)	20 (1.65%)		
Slovenia	2 (0.46%)	2 (0.08%)	441 (36.4%)		
Spain	15 (3.45%)	2,055 (77.7%)	46 (3.79%)		
Switzerland	0 (0.00%)	0 (0.00%)	15 (1.24%)		
The Netherlands	0 (0.00%)	0 (0.00%)	9 (0.74%)		
Turkey	0 (0.00%)	2 (0.08%)	83 (6.84%)		
Ukraine	0 (0.00%)	28 (1.06%)	4 (0.33%)		
United Kingdom	0 (0.00%)	0 (0.00%)	83 (6.84%)		

**Most frequent 1st line treatments**				4,292
Triple-CA/M	4 (0.92%)	1,371 (51.9%)	942 (77.7%)	
Seq-CAT-CAM	369 (84.8%)	6 (0.23%)	1 (0.08%)	
Conco-CAT CAM	4 (0.92%)	900 (34.0%)	3 (0.25%)	
BsQuad-MTcB (including single capsule)	44 (10.1%)	26 (0.98%)	6 (0.49%)	
Quadruple-CAB	0 (0.00%)	141 (5.33%)	0 (0.00%)	
Other[3]	14 (3.22%)	200 (7.56%)	261 (21.5%)	

A: amoxicillin; B: bismuth salts; C: clarithromycin; Conco: concomitant; M: metronidazole; N: total number of cases in year evaluated; n: number of cases in each cluster; OE: omeprazole equivalent; PPI: proton pump inhibitor; Seq, sequential; T: tinidazole; Tc: tetracycline hydrochloride; MTcB was prescribed either in the classical form or as three-in-one single capsule, marketed as Pylera®. [1]Other gastrointestinal symptoms (excluding the most frequent ones such as dyspepsia or heartburn) included nausea, diarrhoea and weight loss. [2]Low dose PPI: 4.5–27 mg omeprazole equivalents, two times per day (i.e., 20 mg omeprazole equivalents, two times per day); standard dose PPI: 32–40 mg omeprazole equivalents, two times per day (i.e., 40 mg omeprazole equivalents, two times per day); high dose PPI: 54–128 mg omeprazole equivalents, two times per day (i.e., 80 mg omeprazole equivalents, two times per day).[3]Other treatments encompassed less than 10% of the remaining prescribed regimens. Statistical significance was set at p-value < 0.05**.**

Table S3. Summary description of patients and variables in each cluster, corresponding to the year 2015.

	1 (n=346)	2 (n=2,656)	3 (n=691)	Overall p-value	N
**Gastrointestinal symptoms**				<0.001	3,693
Absence (none)	344 (99.4%)	2,267 (85.4%)	607 (87.8%)		
Other symptoms[1]	2 (0.58%)	389 (14.6%)	84 (12.2%)		
**Compliance**				0.009	3,693
No (< 90% drug intake)	8 (2.31%)	48 (1.81%)	2 (0.29%)		
Yes (> 90% drug intake)	338 (97.7%)	2,608 (98.2%)	689 (99.7%)		
**Duration (days)**				0.000	3,693
7	0 (0.00%)	15 (0.56%)	672 (97.3%)		
10	338 (97.7%)	1,956 (73.6%)	18 (2.60%)		
14	8 (2.31%)	685 (25.8%)	1 (0.14%)		
**Dose of PPI (mg OE)[2]**				<0.001	3,693
Low	40 (11.6%)	1,092 (41.1%)	494 (71.5%)		
Standard	9 (2.60%)	803 (30.2%)	91 (13.2%)		
High	297 (85.8%)	761 (28.7%)	106 (15.3%)		
**Country**					3,693
Belgium	0 (0.00%)	5 (0.19%)	0 (0.00%)		
Croatia	0 (0.00%)	5 (0.19%)	0 (0.00%)		
Germany	0 (0.00%)	0 (0.00%)	16 (2.32%)		
Greece	10 (2.89%)	58 (2.18%)	0 (0.00%)		
Hungary	0 (0.00%)	22 (0.83%)	18 (2.60%)		
Ireland	0 (0.00%)	27 (1.02%)	0 (0.00%)		
Israel	3 (0.87%)	3 (0.11%)	0 (0.00%)		
Italy	288 (83.2%)	0 (0.00%)	1 (0.14%)		
Latvia	0 (0.00%)	71 (2.67%)	48 (6.95%)		
Lithuania	0 (0.00%)	0 (0.00%)	48 (6.95%)		
Norway	0 (0.00%)	107 (4.03%)	10 (1.45%)		
Portugal	12 (3.47%)	6 (0.23%)	0 (0.00%)		
Romania	0 (0.00%)	0 (0.00%)	29 (4.20%)		
Russia	0 (0.00%)	356 (13.4%)	30 (4.34%)		
Slovenia	8 (2.31%)	0 (0.00%)	371 (53.7%)		
Spain	23 (6.65%)	1,956 (73.6%)	25 (3.62%)		
Switzerland	0 (0.00%)	10 (0.38%)	0 (0.00%)		
Turkey	2 (0.58%)	0 (0.00%)	66 (9.55%)		
Ukraine	0 (0.00%)	30 (1.13%)	0 (0.00%)		
United Kingdom	0 (0.00%)	0 (0.00%)	29 (4.20%)		
**Most frequent 1st line treatments**					3,693
Triple-CA/M	0 (0.00%)	1,118 (42.1%)	600 (86.8%)		
Seq-CAT-CAM	257 (74.3%)	3 (0.11%)	0 (0.00%)		
Conco-CAT CAM	74 (21.4%)	933 (35.1%)	0 (0.00%)		

BsQuad-MTcB (including single capsule)	7 (2.02%)	29 (1.09%)	1 (0.14%)
Quadruple-CAB	0 (0.00%)	208 (7.83%)	0 (0.00%)
Other[3]	8 (2.31%)	365 (13.7%)	90 (13.0%)

A: amoxicillin; B: bismuth salts; C: clarithromycin; Conco: concomitant; M: metronidazole; N: total number of cases in year evaluated; n: number of cases in each cluster; OE: omeprazole equivalent; PPI: proton pump inhibitor; Seq, sequential; T: tinidazole; Tc: tetracycline hydrochloride; MTcB was prescribed either in the classical form or as three-in-one single capsule, marketed as Pylera®. [1]Other gastrointestinal symptoms (excluding the most frequent ones such as dyspepsia or heartburn) included nausea, diarrhoea and weight loss. [2]Low dose PPI: 4.5–27 mg omeprazole equivalents, two times per day (i.e., 20 mg omeprazole equivalents, two times per day); standard dose PPI: 32–40 mg omeprazole equivalents, two times per day (i.e., 40 mg omeprazole equivalents, two times per day); high dose PPI: 54–128 mg omeprazole equivalents, two times per day (i.e., 80 mg omeprazole equivalents, two times per day).[3]Other treatments encompassed less than 10% of the remaining prescribed regimens. Statistical significance was set at p-value < 0.05**.**

Table S4. Summary description of patients and variables in each, corresponding to the year 2016.

	1 (n=2,523)	2 (n=1,152)	3 (n=675)	Overall p-value	N
**Gastrointestinal symptoms**				<0.001	4,350
Absence (none)	2,094 (83.0%)	1,074 (93.2%)	599 (88.7%)		
Other symptoms[1]	429 (17.0%)	78 (6.77%)	76 (11.3%)		
**Compliance**				0.053	4,350
No (< 90% drug intake)	46 (1.82%)	15 (1.30%)	4 (0.59%)		
Yes (> 90% drug intake)	2,477 (98.2%)	1,137 (98.7%)	671 (99.4%)		
**Duration (days)**				0.000	4,350
7	0 (0.00%)	39 (3.39%)	543 (80.4%)		
10	1361 (53.9%)	806 (70.0%)	64 (9.48%)		
14	1162 (46.1%)	307 (26.6%)	68 (10.1%)		
**Dose of PPI (mg OE)[2]**				<0.001	4,350
Low	857 (34.0%)	490 (42.5%)	333 (49.3%)		
Standard	398 (15.8%)	527 (45.7%)	83 (12.3%)		
High	1,268 (50.3%)	135 (11.7%)	259 (38.4%)		
**Country**					4,350
Croatia	0 (0.00%)	13 (1.13%)	0 (0.00%)		
Czech Republic	0 (0.00%)	2 (0.17%)	4 (0.59%)		
France	5 (0.20%)	1 (0.09%)	0 (0.00%)		
Germany	16 (0.63%)	0 (0.00%)	23 (3.41%)		
Greece	73 (2.89%)	5 (0.43%)	0 (0.00%)		
Hungary	0 (0.00%)	29 (2.52%)	0 (0.00%)		
Ireland	0 (0.00%)	0 (0.00%)	28 (4.15%)		
Israel	2 (0.08%)	35 (3.04%)	20 (2.96%)		
Italy	294 (11.7%)	47 (4.08%)	0 (0.00%)		
Latvia	0 (0.00%)	2 (0.17%)	84 (12.4%)		
Lithuania	0 (0.00%)	1 (0.09%)	57 (8.44%)		
Norway	0 (0.00%)	136 (11.8%)	2 (0.30%)		
Portugal	0 (0.00%)	14 (1.22%)	0 (0.00%)		
Romania	0 (0.00%)	3 (0.26%)	1 (0.15%)		
Russia	5 (0.20%)	799 (69.4%)	30 (4.44%)		
Serbia	8 (0.32%)	3 (0.26%)	0 (0.00%)		
Slovenia	0 (0.00%)	0 (0.00%)	331 (49.0%)		
Spain	2,041 (80.9%)	61 (5.30%)	19 (2.81%)		
Switzerland	0 (0.00%)	0 (0.00%)	7 (1.04%)		
Turkey	0 (0.00%)	0 (0.00%)	38 (5.63%)		
Ukraine	79 (3.13%)	0 (0.00%)	1 (0.15%)		
United Kingdom	0 (0.00%)	1 (0.09%)	30 (4.44%)		
**Most frequent 1st line treatments**					4,350
Triple-CA/M	345 (13.7%)	434 (37.7%)	624 (92.4%)		

Seq-CAT-CAM	0 (0.00%)	79 (6.86%)	0 (0.00%)
Conco-CAT CAM	881 (34.9%)	10 (0.87%)	1 (0.15%)
BsQuad-MTcB (including single capsule)	652 (25.8%)	3 (0.26%)	0 (0.00%)
Quadruple-CAB	594 (23.5%)	221 (19.2%)	0 (0.00%)
Other[3]	51 (2.02%)	405 (35.2%)	50 (7.41%)

A: amoxicillin; B: bismuth salts; C: clarithromycin; Conco: concomitant; M: metronidazole; N: total number of cases in year evaluated; n: number of cases in each cluster; OE: omeprazole equivalent; PPI: proton pump inhibitor; Seq, sequential; T: tinidazole; Tc: tetracycline hydrochloride; MTcB was prescribed either in the classical form or as three-in-one single capsule, marketed as Pylera®. [1]Other gastrointestinal symptoms (excluding the most frequent ones such as dyspepsia or heartburn) included nausea, diarrhoea and weight loss. [2]Low dose PPI: 4.5–27 mg omeprazole equivalents, two times per day (i.e., 20 mg omeprazole equivalents, two times per day); standard dose PPI: 32–40 mg omeprazole equivalents, two times per day (i.e., 40 mg omeprazole equivalents, two times per day); high dose PPI: 54–128 mg omeprazole equivalents, two times per day (i.e., 80 mg omeprazole equivalents, two times per day).[3]Other treatments encompassed less than 10% of the remaining prescribed regimens. Statistical significance was set at p-value <**.**

Table S5. Summary description of patients and variables in each cluster, corresponding to the year 2017.

	1 (n=323)	2 (n=1,969)	3 (n=1,373)	Overall p-value	N
**Gastrointestinal symptoms**				<0.001	3,665
Absence (none)	319 (98.8%)	1,773 (90.0%)	1,185 (86.3%)		
Other symptoms[1]	4 (1.24%)	196 (9.95%)	188 (13.7%)		
**Compliance**				<0.001	3,665
No (< 90% drug intake)	0 (0.00%)	35 (1.78%)	7 (0.51%)		
Yes (> 90% drug intake)	323 (100%)	1,934 (98.2%)	1,366 (99.5%)		
**Duration (days)**				<0.001	3,665
7	0 (0.00%)	1 (0.05%)	173 (12.6%)		
10	322 (99.7%)	1,150 (58.4%)	548 (39.9%)		
14	1 (0.31%)	818 (41.5%)	652 (47.5%)		
**Dose of PPI (mg OE)[2]**				<0.001	3,665
Low	308 (95.4%)	817 (41.5%)	500 (36.4%)		
Standard	3 (0.93%)	368 (18.7%)	428 (31.2%)		
High	12 (3.72%)	784 (39.8%)	445 (32.4%)		
**Country**					3,665
Croatia	0 (0.00%)	0 (0.00%)	7 (0.51%)		
Czech Republic	0 (0.00%)	0 (0.00%)	3 (0.22%)		
France	2 (0.62%)	1 (0.05%)	2 (0.15%)		
Germany	0 (0.00%)	24 (1.22%)	15 (1.09%)		
Greece	0 (0.00%)	7 (0.36%)	23 (1.68%)		
Hungary	0 (0.00%)	3 (0.15%)	25 (1.82%)		
Ireland	0 (0.00%)	0 (0.00%)	56 (4.08%)		
Israel	0 (0.00%)	3 (0.15%)	8 (0.58%)		
Italy	316 (97.8%)	105 (5.33%)	20 (1.46%)		
Latvia	0 (0.00%)	1 (0.05%)	24 (1.75%)		
Lithuania	0 (0.00%)	0 (0.00%)	85 (6.19%)		
Norway	0 (0.00%)	0 (0.00%)	111 (8.08%)		
Poland	1 (0.31%)	0 (0.00%)	0 (0.00%)		
Portugal	2 (0.62%)	61 (3.10%)	4 (0.29%)		
Russia	2 (0.62%)	4 (0.20%)	624 (45.4%)		
Slovenia	0 (0.00%)	0 (0.00%)	277 (20.2%)		
Spain	0 (0.00%)	1,760 (89.4%)	38 (2.77%)		
Ukraine	0 (0.00%)	0 (0.00%)	46 (3.35%)		
United Kingdom	0 (0.00%)	0 (0.00%)	5 (0.36%)		
**Most frequent 1st line treatments**					3,665
Triple-CA/M	10 (3.10%)	95 (4.82%)	856 (62.3%)		
Seq-CAT-CAM	287 (88.9%)	0 (0.00%)	1 (0.07%)		
Conco-CAT CAM	4 (1.24%)	758 (38.5%)	1 (0.07%)		

BsQuad-MTcB (including single capsule)	17 (5.26%)	851 (43.2%)	4 (0.29%)
Quadruple-CAB	0 (0.00%)	218 (11.1%)	213 (15.5%)
Other[3]	5 (1.55%)	47 (2.39%)	298 (21.7%)

A: amoxicillin; B: bismuth salts; C: clarithromycin; Conco: concomitant; M: metronidazole; N: total number of cases in year evaluated; n: number of cases in each cluster; OE: omeprazole equivalent; PPI: proton pump inhibitor; Seq, sequential; T: tinidazole; Tc: tetracycline hydrochloride; MTcB was prescribed either in the classical form or as three-in-one single capsule, marketed as Pylera®. [1]Other gastrointestinal symptoms (excluding the most frequent ones such as dyspepsia or heartburn) included nausea, diarrhoea and weight loss. [2]Low dose PPI: 4.5–27 mg omeprazole equivalents, two times per day (i.e., 20 mg omeprazole equivalents, two times per day); standard dose PPI: 32–40 mg omeprazole equivalents, two times per day (i.e., 40 mg omeprazole equivalents, two times per day); high dose PPI: 54–128 mg omeprazole equivalents, two times per day (i.e., 80 mg omeprazole equivalents, two times per day).[3]Other treatments encompassed less than 10% of the remaining prescribed regimens. Statistical significance was set at p-value <**.**

Table S6. Summary description of patients and variables in each, corresponding to the year 2018.

	1 (n=1,974)	2 (n=1,342)	3 (n=352)	Overall p-value	N
**Gastrointestinal symptoms**				<0.001	3,668
Absence (none)	1,837 (93.1%)	1,139 (84.9%)	349 (99.1%)		
Other symptoms[1]	137 (6.94%)	203 (15.1%)	3 (0.85%)		
**Compliance**				0.071	3,668
No (< 90% drug intake)	22 (1.11%)	23 (1.71%)	1 (0.28%)		
Yes (> 90% drug intake)	1,952 (98.9%)	1,319 (98.3%)	351 (99.7%)		
**Duration (days)**				<0.001	3,668
7	67 (3.39%)	0 (0.00%)	0 (0.00%)		
10	578 (29.3%)	820 (61.1%)	348 (98.9%)		
14	1,329 (67.3%)	522 (38.9%)	4 (1.14%)		
**Dose of PPI (mg OE)[2]**				<0.001	3,668
Low	345 (17.5%)	396 (29.5%)	339 (96.3%)		
Standard	1,097 (55.6%)	234 (17.4%)	5 (1.42%)		
High	532 (27.0%)	712 (53.1%)	8 (2.27%)		
**Country**					3,668
Croatia	45 (2.28%)	10 (0.75%)	3 (0.85%)		
Czech Republic	8 (0.41%)	1 (0.07%)	0 (0.00%)		
Germany	6 (0.30%)	8 (0.60%)	0 (0.00%)		
Greece	0 (0.00%)	24 (1.79%)	0 (0.00%)		
Hungary	33 (1.67%)	1 (0.07%)	0 (0.00%)		
Ireland	110 (5.57%)	0 (0.00%)	0 (0.00%)		
Israel	3 (0.15%)	6 (0.45%)	0 (0.00%)		
Italy	20 (1.01%)	86 (6.41%)	340 (96.6%)		
Latvia	20 (1.01%)	0 (0.00%)	0 (0.00%)		
Lithuania	60 (3.04%)	0 (0.00%)	0 (0.00%)		
Norway	2 (0.10%)	0 (0.00%)	0 (0.00%)		
Poland	0 (0.00%)	8 (0.60%)	5 (1.42%)		
Portugal	5 (0.25%)	56 (4.17%)	2 (0.57%)		
Russia	1,375 (69.7%)	6 (0.45%)	2 (0.57%)		
Serbia	3 (0.15%)	2 (0.15%)	0 (0.00%)		
Slovenia	270 (13.7%)	0 (0.00%)	0 (0.00%)		
Spain	9 (0.46%)	1,134 (84.5%)	0 (0.00%)		
United Kingdom	5 (0.25%)	0 (0.00%)	0 (0.00%)		
**Most frequent 1st line treatments**					3,668
Triple-CA/M	887 (44.9%)	20 (1.49%)	4 (1.14%)		
Seq-CAT-CAM	3 (0.15%)	0 (0.00%)	291 (82.7%)		
Conco-CAT CAM	2 (0.10%)	361 (26.9%)	0 (0.00%)		
BsQuad-MTcB (including single capsule)	16 (0.81%)	708 (52.8%)	42 (11.9%)		

Quadruple-CAB	406 (20.6%)	208 (15.5%)	0 (0.00%)	
Other[3]	660 (33.4%)	45 (3.35%)	15 (4.26%)	

A: amoxicillin; B: bismuth salts; C: clarithromycin; Conco: concomitant; M: metronidazole; N: total number of cases in year evaluated; n: number of cases in each cluster; OE: omeprazole equivalent; PPI: proton pump inhibitor; Seq, sequential; T: tinidazole; Tc: tetracycline hydrochloride; MTcB was prescribed either in the classical form or as three-in-one single capsule, marketed as Pylera®. [1]Other gastrointestinal symptoms (excluding the most frequent ones such as dyspepsia or heartburn) included nausea, diarrhoea and weight loss. [2]Low dose PPI: 4.5–27 mg omeprazole equivalents, two times per day (i.e., 20 mg omeprazole equivalents, two times per day); standard dose PPI: 32–40 mg omeprazole equivalents, two times per day (i.e., 40 mg omeprazole equivalents, two times per day); high dose PPI: 54–128 mg omeprazole equivalents, two times per day (i.e., 80 mg omeprazole equivalents, two times per day).[3]Other treatments encompassed less than 10% of the remaining prescribed regimens. Statistical significance was set at p-value < 0.05**.**

Table S7. Summary description of patients and variables in each cluster they belong, corresponding to the year 2019.

	1 (n=1,225)	2 (n=721)	3 (n=1,749)	Overall p-value	N
**Gastrointestinal symptoms**				<0.001	3,695
Absence (none)	952 (77.7%)	676 (93.8%)	1,551 (88.7%)		
Other symptoms[1]	273 (22.3%)	45 (6.24%)	198 (11.3%)		
**Compliance**				0.097	3,695
No (< 90% drug intake)	17 (1.39%)	4 (0.55%)	29 (1.66%)		
Yes (> 90% drug intake)	1,208 (98.6%)	717 (99.4%)	1,720 (98.3%)		
**Duration (days)**				<0.001	3,695
7	6 (0.49%)	72 (9.99%)	17 (0.97%)		
10	157 (12.8%)	123 (17.1%)	1,192 (68.2%)		
14	1,062 (86.7%)	526 (73.0%)	540 (30.9%)		
**Dose of PPI (mg OE)[2]**				<0.001	3,695
Low	270 (22.0%)	133 (18.4%)	869 (49.7%)		
Standard	741 (60.5%)	31 (4.30%)	219 (12.5%)		
High	214 (17.5%)	557 (77.3%)	661 (37.8%)		
**Country**					3,695
Bulgaria	2 (0.16%)	0 (0.00%)	0 (0.00%)		
Croatia	16 (1.31%)	4 (0.55%)	114 (6.52%)		
Czech Republic	2 (0.16%)	5 (0.69%)	46 (2.63%)		
Germany	1 (0.08%)	0 (0.00%)	5 (0.29%)		
Greece	0 (0.00%)	0 (0.00%)	34 (1.94%)		
Hungary	22 (1.80%)	0 (0.00%)	0 (0.00%)		
Ireland	0 (0.00%)	228 (31.6%)	0 (0.00%)		
Israel	0 (0.00%)	0 (0.00%)	2 (0.11%)		
Italy	0 (0.00%)	7 (0.97%)	376 (21.5%)		
Latvia	0 (0.00%)	102 (14.1%)	0 (0.00%)		
Lithuania	6 (0.49%)	80 (11.1%)	1 (0.06%)		
Poland	0 (0.00%)	0 (0.00%)	31 (1.77%)		
Portugal	6 (0.49%)	8 (1.11%)	130 (7.43%)		
Russia	1,124 (91.8%)	4 (0.55%)	2 (0.11%)		
Serbia	2 (0.16%)	1 (0.14%)	41 (2.34%)		
Slovenia	0 (0.00%)	270 (37.4%)	0 (0.00%)		
Spain	43 (3.51%)	2 (0.28%)	967 (55.3%)		
Ukraine	1 (0.08%)	0 (0.00%)	0 (0.00%)		
United Kingdom	0 (0.00%)	10 (1.39%)	0 (0.00%)		
**Most frequent 1st line treatments**					3,695
Triple-CA/M	462 (37.7%)	693 (96.1%)	80 (4.57%)		
Seq-CAT-CAM	1 (0.08%)	0 (0.00%)	199 (11.4%)		
Conco-CAT CAM	26 (2.12%)	0 (0.00%)	473 (27.0%)		

BsQuad-MTcB (including single capsule)	39 (3.18%)	2 (0.28%)	823 (47.1%)	
Quadruple-CAB	361 (29.5%)	0 (0.00%)	8 (0.46%)	
Other[3]	336 (27.4%)	26 (3.61%)	166 (9.49%)	

A: amoxicillin; B: bismuth salts; C: clarithromycin; Conco: concomitant; M: metronidazole; N: total number of cases in year evaluated; n: number of cases in each cluster; OE: omeprazole equivalent; PPI: proton pump inhibitor; Seq, sequential; T: tinidazole; Tc: tetracycline hydrochloride; MTcB was prescribed either in the classical form or as three-in-one single capsule, marketed as Pylera®. [1]Other gastrointestinal symptoms (excluding the most frequent ones such as dyspepsia or heartburn) included nausea, diarrhoea and weight loss. [2]Low dose PPI: 4.5–27 mg omeprazole equivalents, two times per day (i.e., 20 mg omeprazole equivalents, two times per day); standard dose PPI: 32–40 mg omeprazole equivalents, two times per day (i.e., 40 mg omeprazole equivalents, two times per day); high dose PPI: 54–128 mg omeprazole equivalents, two times per day (i.e., 80 mg omeprazole equivalents, two times per day).[3]Other treatments encompassed less than 10% of the remaining prescribed regimens. Statistical significance was set at p-<0.05**.**

Table S8. Summary description of patients and variables in each cluster, corresponding to the year 2020.

	1 (n=1,783)	2 (n=585)	3 (n=724)	Overall p-value	N
**Gastrointestinal symptoms**				0.011	3,092
Absence (none)	1,575 (88.3%)	536 (91.6%)	625 (86.3%)		
Other symptoms[1]	208 (11.7%)	49 (8.38%)	99 (13.7%)		
**Compliance**				0.019	3,092
No (< 90% drug intake)	36 (2.02%)	2 (0.34%)	11 (1.52%)		
Yes (> 90% drug intake)	1,747 (98.0%)	583 (99.7%)	713 (98.5%)		
**Duration (days)**				<0.001	3,092
7	5 (0.28%)	108 (18.5%)	7 (0.97%)		
10	1,208 (67.8%)	113 (19.3%)	78 (10.8%)		
14	570 (32.0%)	364 (62.2%)	639 (88.3%)		
**Dose of PPI (mg OE)[2]**				<0.001	3,092
Low	997 (55.9%)	170 (29.1%)	90 (12.4%)		
Standard	353 (19.8%)	43 (7.35%)	412 (56.9%)		
High	433 (24.3%)	372 (63.6%)	222 (30.7%)		
**Country**					3,092
Bulgaria	0 (0.00%)	0 (0.00%)	5 (0.69%)		
Croatia	0 (0.00%)	2 (0.34%)	31 (4.28%)		
Czech Republic	11 (0.62%)	89 (15.2%)	7 (0.97%)		
Germany	23 (1.29%)	8 (1.37%)	1 (0.14%)		
Greece	24 (1.35%)	0 (0.00%)	7 (0.97%)		
Hungary	0 (0.00%)	1 (0.17%)	23 (3.18%)		
Ireland	0 (0.00%)	138 (23.6%)	0 (0.00%)		
Israel	32 (1.79%)	0 (0.00%)	6 (0.83%)		
Italy	254 (14.2%)	4 (0.68%)	2 (0.28%)		
Latvia	0 (0.00%)	42 (7.18%)	0 (0.00%)		
Lithuania	2 (0.11%)	86 (14.7%)	2 (0.28%)		
Poland	41 (2.30%)	0 (0.00%)	12 (1.66%)		
Portugal	24 (1.35%)	3 (0.51%)	0 (0.00%)		
Russia	12 (0.67%)	14 (2.39%)	594 (82.0%)		
Serbia	18 (1.01%)	0 (0.00%)	8 (1.10%)		
Slovenia	0 (0.00%)	164 (28.0%)	0 (0.00%)		
Spain	1,342 (75.3%)	3 (0.51%)	25 (3.45%)		
The Netherlands	0 (0.00%)	3 (0.51%)	0 (0.00%)		
Ukraine	0 (0.00%)	0 (0.00%)	1 (0.14%)		
United Kingdom	0 (0.00%)	28 (4.79%)	0 (0.00%)		
**Most frequent 1st line treatments**					3,092
Triple-CA/M	78 (4.37%)	521 (89.1%)	41 (5.66%)		
Seq-CAT-CAM	126 (7.07%)	0 (0.00%)	0 (0.00%)		
Conco-CAT CAM	620 (34.8%)	2 (0.34%)	6 (0.83%)		

BsQuad-MTcB (including single capsule)	882 (49.5%)	5 (0.85%)	5 (0.69%)
Quadruple-CAB	10 (0.56%)	4 (0.68%)	229 (31.6%)
Other[3]	67 (3.76%)	53 (9.06%)	443 (61.2%)

A: amoxicillin; B: bismuth salts; C: clarithromycin; Conco: concomitant; M: metronidazole; N: total number of cases in year evaluated; n: number of cases in each cluster; OE: omeprazole equivalent; PPI: proton pump inhibitor; Seq, sequential; T: tinidazole; Tc: tetracycline hydrochloride; MTcB was prescribed either in the classical form or as three-in-one single capsule, marketed as Pylera®. [1]Other gastrointestinal symptoms (excluding the most frequent ones such as dyspepsia or heartburn) included nausea, diarrhoea and weight loss. [2]Low dose PPI: 4.5–27 mg omeprazole equivalents, two times per day (i.e., 20 mg omeprazole equivalents, two times per day); standard dose PPI: 32–40 mg omeprazole equivalents, two times per day (i.e., 40 mg omeprazole equivalents, two times per day); high dose PPI: 54–128 mg omeprazole equivalents, two times per day (i.e., 80 mg omeprazole equivalents, two times per day).[3]Other treatments encompassed less than 10% of the remaining prescribed regimens. Statistical significance was set at p-value < 0.05**.**

Table S9. Summary description of patients and variables in each cluster, corresponding to the year 2021.

	1 (n=310)	2 (n=1,882)	3 (n=1,826)	Overall p-value	N
**Gastrointestinal symptoms**				<0.001	4,018
Absence (none)	305 (98.4%)	1,652 (87.8%)	1,558 (85.3%)		
Other symptoms[1]	5 (1.61%)	230 (12.2%)	268 (14.7%)		
**Compliance**				0.034	4,018
No (< 90% drug intake)	2 (0.65%)	36 (1.91%)	18 (0.99%)		
Yes (> 90% drug intake)	308 (99.4%)	1,846 (98.1%)	1,808 (99.0%)		
**Duration (days)**				<0.001	4,018
7	0 (0.00%)	1 (0.05%)	102 (5.59%)		
10	305 (98.4%)	1,158 (61.5%)	251 (13.7%)		
14	5 (1.61%)	723 (38.4%)	1,473 (80.7%)		
**Dose of PPI (mg OE)[2]**				<0.001	4,018
Low	271 (87.4%)	873 (46.4%)	406 (22.2%)		
Standard	1 (0.32%)	432 (23.0%)	765 (41.9%)		
High	38 (12.3%)	577 (30.7%)	655 (35.9%)		
**Country**					4,018
Bulgaria	0 (0.00%)	1 (0.05%)	27 (1.48%)		
Croatia	0 (0.00%)	0 (0.00%)	46 (2.52%)		
Czech Republic	7 (2.26%)	1 (0.05%)	96 (5.26%)		
Germany	0 (0.00%)	46 (2.44%)	9 (0.49%)		
Greece	0 (0.00%)	77 (4.09%)	3 (0.16%)		
Hungary	0 (0.00%)	0 (0.00%)	30 (1.64%)		
Ireland	0 (0.00%)	0 (0.00%)	133 (7.28%)		
Israel	0 (0.00%)	3 (0.16%)	1 (0.05%)		
Italy	303 (97.7%)	24 (1.28%)	14 (0.77%)		
Latvia	0 (0.00%)	0 (0.00%)	45 (2.46%)		
Lithuania	0 (0.00%)	0 (0.00%)	98 (5.37%)		
Poland	0 (0.00%)	47 (2.50%)	11 (0.60%)		
Portugal	0 (0.00%)	27 (1.43%)	2 (0.11%)		
Russia	0 (0.00%)	3 (0.16%)	966 (52.9%)		
Serbia	0 (0.00%)	38 (2.02%)	60 (3.29%)		
Slovenia	0 (0.00%)	1 (0.05%)	179 (9.80%)		
Spain	0 (0.00%)	1,569 (83.4%)	61 (3.34%)		
Switzerland	0 (0.00%)	24 (1.28%)	0 (0.00%)		
The Netherlands	0 (0.00%)	21 (1.12%)	0 (0.00%)		
Ukraine	0 (0.00%)	0 (0.00%)	9 (0.49%)		
United Kingdom	0 (0.00%)	0 (0.00%)	36 (1.97%)		

**Most frequent 1st line treatments**				4,018
Triple-CA/M	16 (5.16%)	58 (3.08%)	623 (34.1%)	
Seq-CAT-CAM	189 (61.0%)	0 (0.00%)	0 (0.00%)	
Conco-CAT CAM	0 (0.00%)	779 (41.4%)	8 (0.44%)	
BsQuad-MTcB (including single capsule)	57 (18.4%)	996 (52.9%)	15 (0.82%)	
Quadruple-CAB	0 (0.00%)	3 (0.16%)	292 (16.0%)	
Other[3]	48 (15.5%)	46 (2.44%)	888 (48.6%)	

A: amoxicillin; B: bismuth salts; C: clarithromycin; Conco: concomitant; M: metronidazole; N: total number of cases in year evaluated; n: number of cases in each cluster; OE: omeprazole equivalent; PPI: proton pump inhibitor; Seq, sequential; T: tinidazole; Tc: tetracycline hydrochloride; MTcB was prescribed either in the classical form or as three-in-one single capsule, marketed as Pylera®. [1]Other gastrointestinal symptoms (excluding the most frequent ones such as dyspepsia or heartburn) included nausea, diarrhoea and weight loss. [2]Low dose PPI: 4.5–27 mg omeprazole equivalents, two times per day (i.e., 20 mg omeprazole equivalents, two times per day); standard dose PPI: 32–40 mg omeprazole equivalents, two times per day (i.e., 40 mg omeprazole equivalents, two times per day); high dose PPI: 54–128 mg omeprazole equivalents, two times per day (i.e., 80 mg omeprazole equivalents, two times per day).[3]Other treatments encompassed less than 10% of the remaining prescribed regimens. Statistical significance was set at p-value < 0.05.

Figure S1. Random Forest variable importance based on mean decrease accuracy for the first six variables (year 2013)

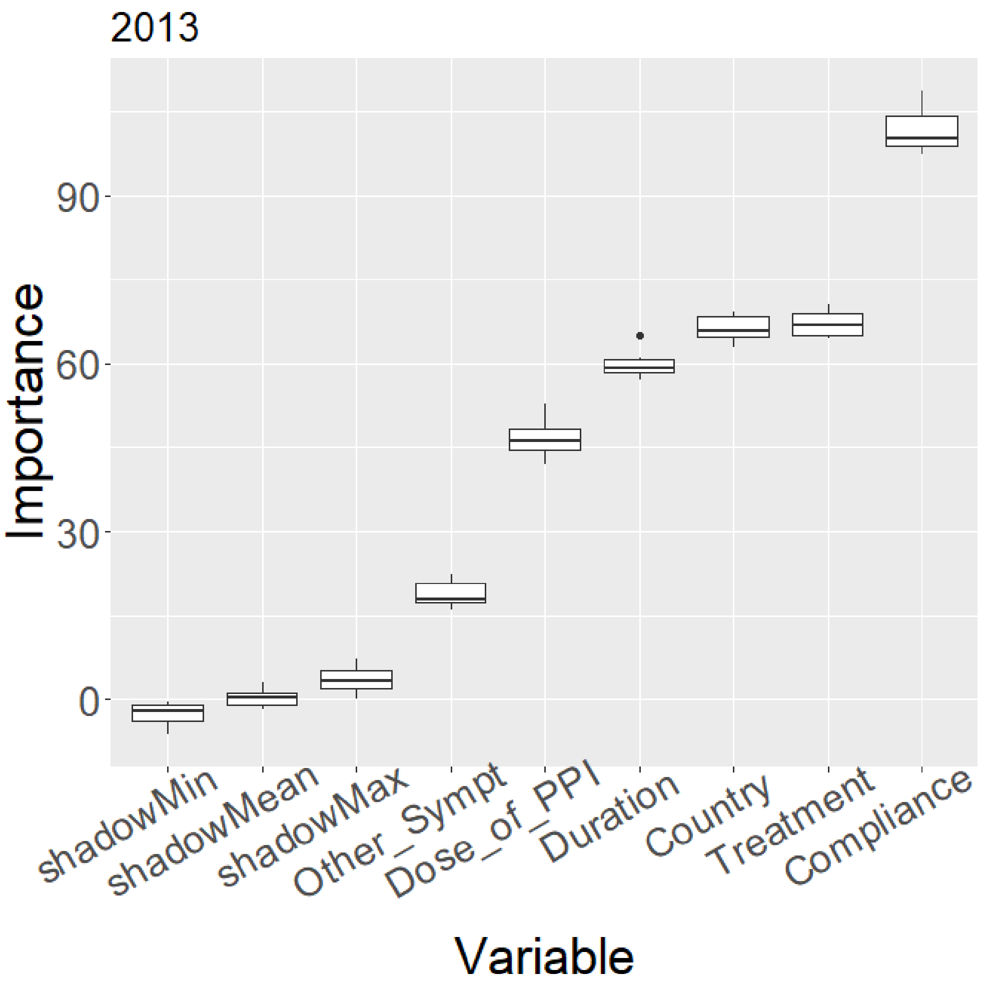

Compliance, defined as 1: yes with >90% drug intake or 0: no with <90% drug intake; Dose of_PPI, defined as low dose PPI: 4.5 to 27 mg OE b.i.d; standard dose PPI: 32 to 40 mg OE b.i.d; high-dose PPI: 54 to 128 mg OE b.i.d; Duration, defined as a duration of treatment of 7, 10 or 14 days; Other_Sympt, defined as other non-frequent gastrointestinal symptoms (0: absence, 1: presence); Treatment, defined as the most frequent 1st line therapies (as defined in Methods section).

Figure S2. Random Forest variable importance based on mean decrease accuracy for the first six variables (year 2014)

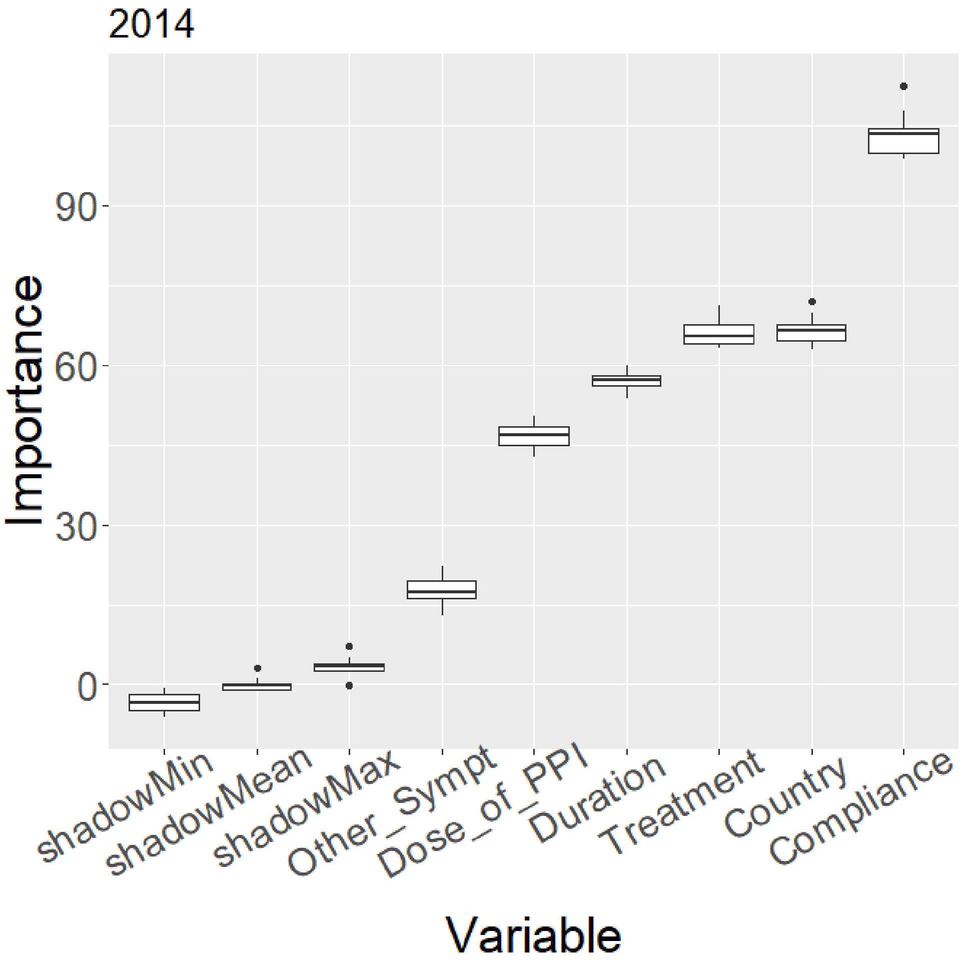

Compliance, defined as 1: yes with >90% drug intake or 0: no with <90% drug intake; Dose of_PPI, defined as low dose PPI: 4.5 to 27 mg OE b.i.d; standard dose PPI: 32 to 40 mg OE b.i.d; high-dose PPI: 54 to 128 mg OE b.i.d; Duration, defined as a duration of treatment of 7, 10 or 14 days; Other_Sympt, defined as other non-frequent gastrointestinal symptoms (0: absence, 1: presence); Treatment, defined as the most frequent 1st line therapies (as defined in Methods section).

Figure S3. Random Forest variable importance based on mean decrease accuracy for the first six variables (year 2015)

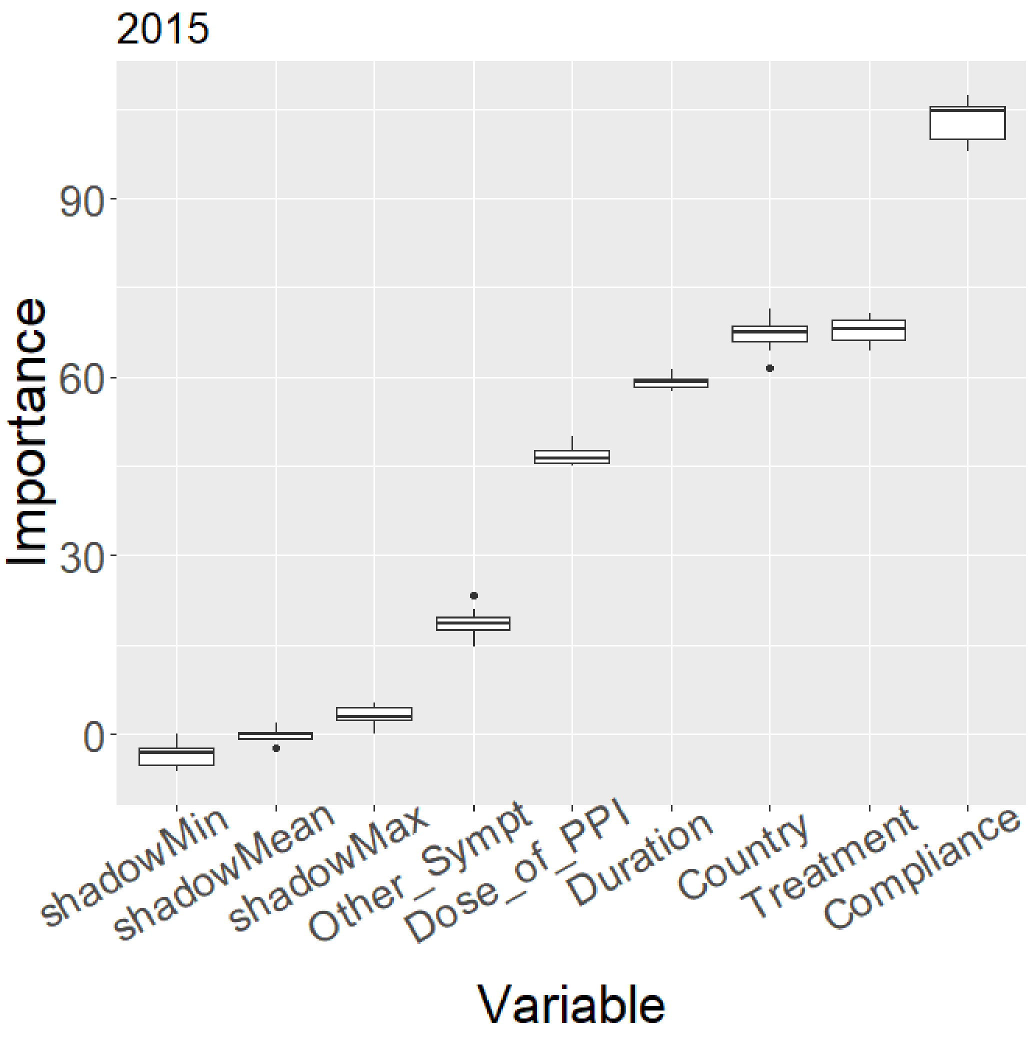

Compliance, defined as 1: yes with >90% drug intake or 0: no with <90% drug intake; Dose of_PPI, defined as low dose PPI: 4.5 to 27 mg OE b.i.d; standard dose PPI: 32 to 40 mg OE b.i.d; high-dose PPI: 54 to 128 mg OE b.i.d; Duration, defined as a duration of treatment of 7, 10 or 14 days; Other_Sympt, defined as other non-frequent gastrointestinal symptoms (0: absence, 1: presence); Treatment, defined as the most frequent 1st line therapies (as defined in Methods section).

Figure S4. Random Forest variable importance based on mean decrease accuracy for the first six variables (year 2016)

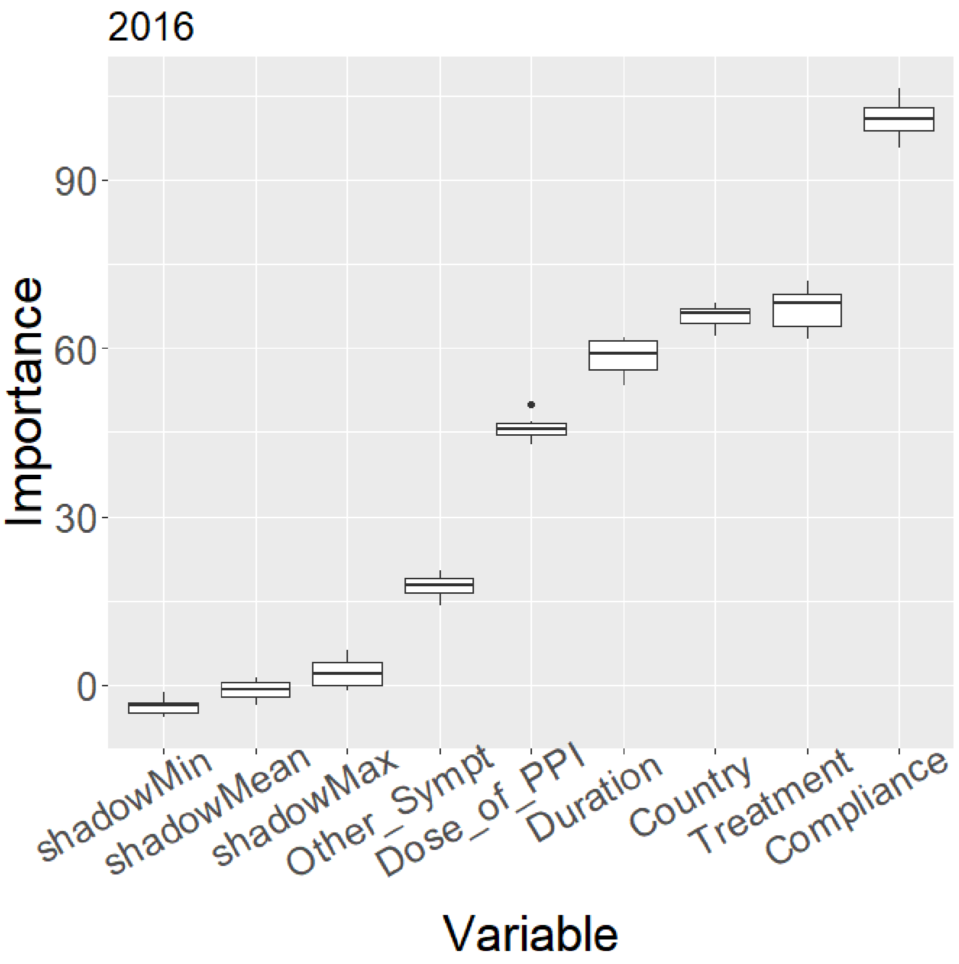

Compliance, defined as 1: yes with >90% drug intake or 0: no with <90% drug intake; Dose of_PPI, defined as low dose PPI: 4.5 to 27 mg OE b.i.d; standard dose PPI: 32 to 40 mg OE b.i.d; high-dose PPI: 54 to 128 mg OE b.i.d; Duration, defined as a duration of treatment of 7, 10 or 14 days; Other_Sympt, defined as other non-frequent gastrointestinal symptoms (0: absence, 1: presence); Treatment, defined as the most frequent 1st line therapies (as defined in Methods section).

Figure S5. Random Forest variable importance based on mean decrease accuracy for the first six variables (year 2017)

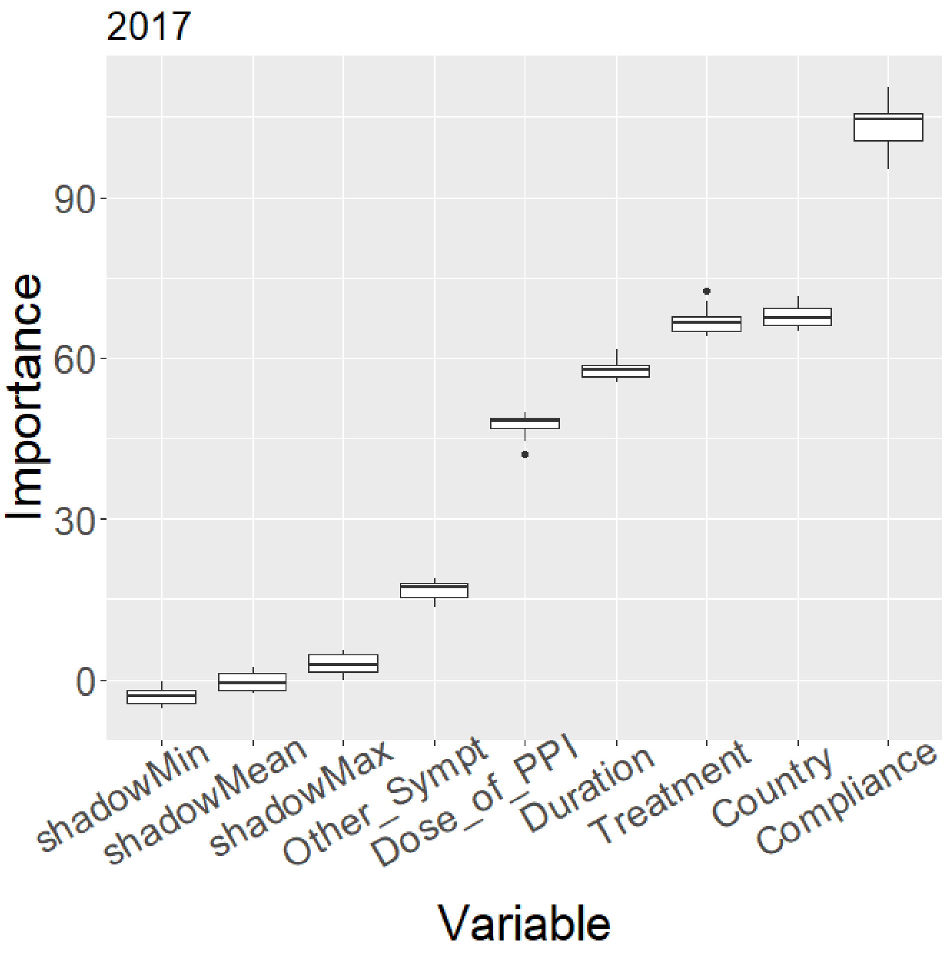

Compliance, defined as 1: yes with >90% drug intake or 0: no with <90% drug intake; Dose of_PPI, defined as low dose PPI: 4.5 to 27 mg OE b.i.d; standard dose PPI: 32 to 40 mg OE b.i.d; high-dose PPI: 54 to 128 mg OE b.i.d; Duration, defined as a duration of treatment of 7, 10 or 14 days; Other_Sympt, defined as other non-frequent gastrointestinal symptoms (0: absence, 1: presence); Treatment, defined as the most frequent 1st line therapies (as defined in Methods section).

Figure S6. Random Forest variable importance based on mean decrease accuracy for the first six variables (year 2018)

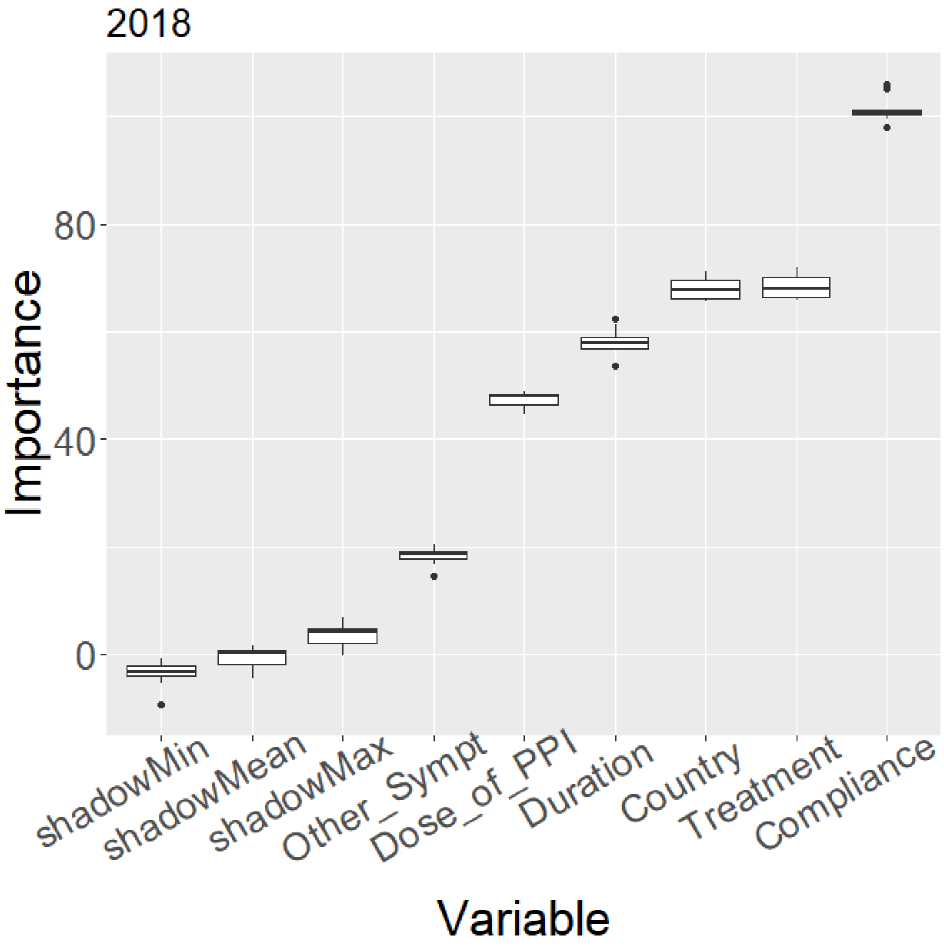

Compliance, defined as 1: yes with >90% drug intake or 0: no with <90% drug intake; Dose of_PPI, defined as low dose PPI: 4.5 to 27 mg OE b.i.d; standard dose PPI: 32 to 40 mg OE b.i.d; high-dose PPI: 54 to 128 mg OE b.i.d; Duration, defined as a duration of treatment of 7, 10 or 14 days; Other_Sympt, defined as other non-frequent gastrointestinal symptoms (0: absence, 1: presence); Treatment, defined as the most frequent 1st line therapies (as defined in Methods section).

Figure S7. Random Forest variable importance based on mean decrease accuracy for the first six variables (year 2019)

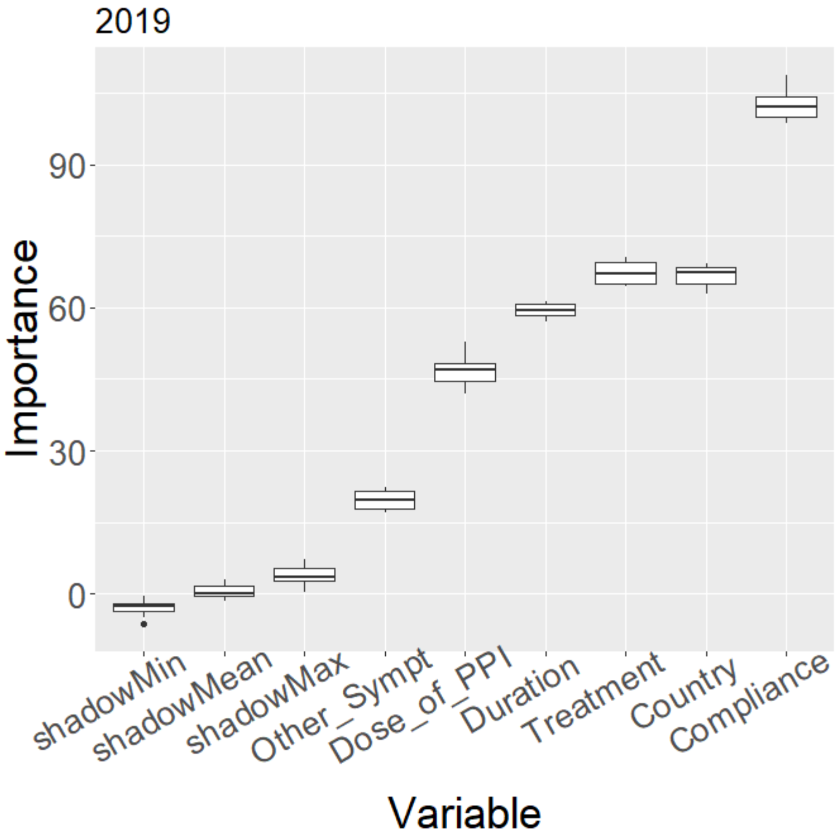

Compliance, defined as 1: yes with >90% drug intake or 0: no with <90% drug intake; Dose of_PPI, defined as low dose PPI: 4.5 to 27 mg OE b.i.d; standard dose PPI: 32 to 40 mg OE b.i.d; high-dose PPI: 54 to 128 mg OE b.i.d; Duration, defined as the duration of treatment of 7, 10 or 14 days; Other_Sympt, defined as other non-frequent gastrointestinal symptoms (0: absence, 1: presence); Treatment, defined as the most frequent 1st line therapies (as defined in Methods section).

Figure S8. Random Forest variable importance based on mean decrease accuracy for the first six variables (year 2020)

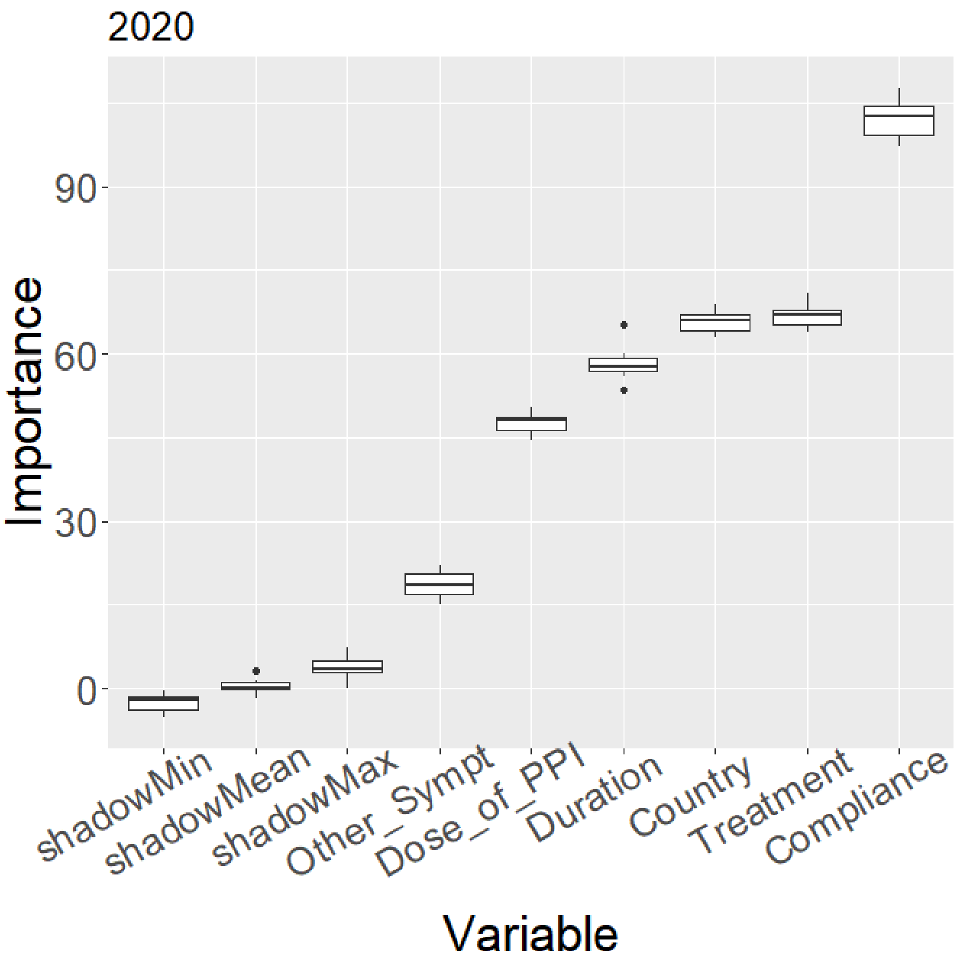

Compliance, defined as 1: yes with >90% drug intake or 0: no with <90% drug intake; Dose of_PPI, defined as low dose PPI: 4.5 to 27 mg OE b.i.d; standard dose PPI: 32 to 40 mg OE b.i.d; high-dose PPI: 54 to 128 mg OE b.i.d; Duration, defined as the duration of treatment of 7, 10 or 14 days; Other_Sympt, defined as other non-frequent gastrointestinal symptoms (0: absence, 1: presence); Treatment, defined as the most frequent 1st line therapies (as defined in Methods section).

Figure S9. Random Forest variable importance based on mean decrease accuracy for the first six variables (year 2021)

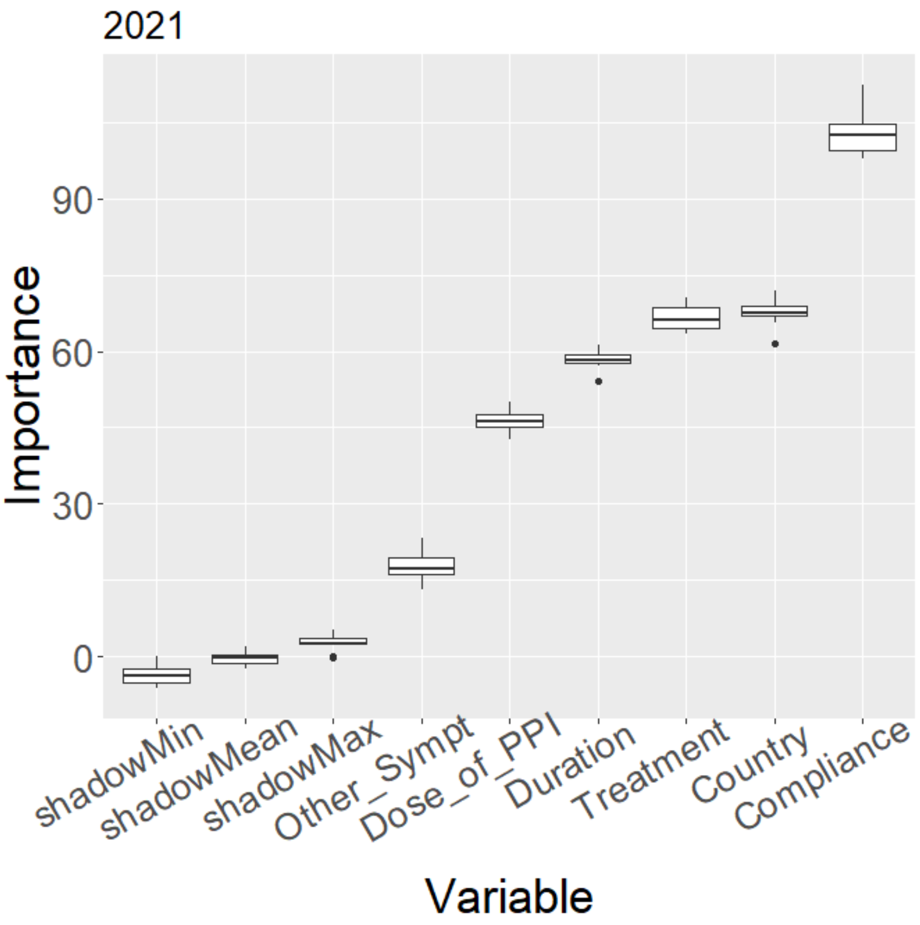

Compliance, defined as 1: yes with >90% drug intake or 0: no with <90% drug intake; Dose of_PPI, defined as low dose PPI: 4.5 to 27 mg OE b.i.d; standard dose PPI: 32 to 40 mg OE b.i.d; high-dose PPI: 54 to 128 mg OE b.i.d; Duration, defined as the duration of treatment of 7, 10 or 14 days; Other_Sympt, defined as other non-frequent gastrointestinal symptoms (0: absence, 1: presence); Treatment, defined as the most frequent 1st line therapies (as defined in Methods section).

Figure S10. Clusters composition in terms of variables' levels in year 2013.

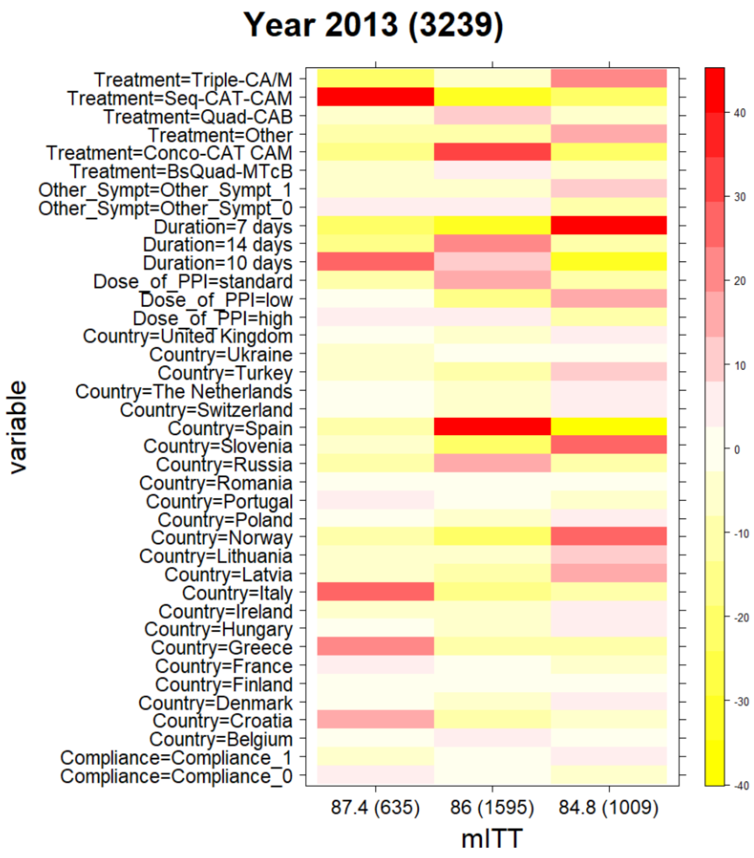

Red colour, high content; yellow colour, ow content.

A: amoxicillin; B: bismuth salts; C: clarithromycin; Conco: concomitant; M: metronidazole; Seq, sequential; T: tinidazole; Tc: tetracycline hydrochloride; MTcB was prescribed either in the classical form or as three-in-one single capsule, marketed as Pylera®. The treatment category "other" encompassed (less than 10% of first-line empirical treatments in Europe) and were mainly quadruple therapy with amoxicillin, metronidazole and bismuth (both in Slovenia and Russia) and quadruple therapy with amoxicillin, clarithromycin and josamycin (in Russia only).

Compliance, defined as 1: yes with >90% drug intake or 0: no with <90% drug intake; Dose of_PPI, defined as low dose PPI: 4.5 to 27 mg OE b.i.d; standard dose PPI: 32 to 40 mg OE b.i.d; high-dose PPI: 54 to 128 mg OE b.i.d; Duration as a duration of treatment of 7, 10 or 14 days; Indication, as ulcer vs dyspepsia; mITT, defined as the modified intention-to-treat; Other_Sympt, defined as other non-frequent gastrointestinal symptoms (0: absence, 1: presence); Sex, as female/male.

In 2013, the highest mITT effectiveness (88%) was observed in cluster #2 (224 cases), mostly with 10-day (83%) and low-dose PPIs (70%) prescriptions, with bismuth quadruple CAB (28% cases) therapy, mostly in Russia (99%).

Among the 3,239 patients included in this analysis, significant (p< 0.001) differences were observed between the 3 clusters in the following variables: presence of other

gastrointestinal symptoms, compliance with treatment, duration of treatment, and country where the treatment was prescribed.

Figure S11. Clusters composition in terms of variables' levels in year 2014.

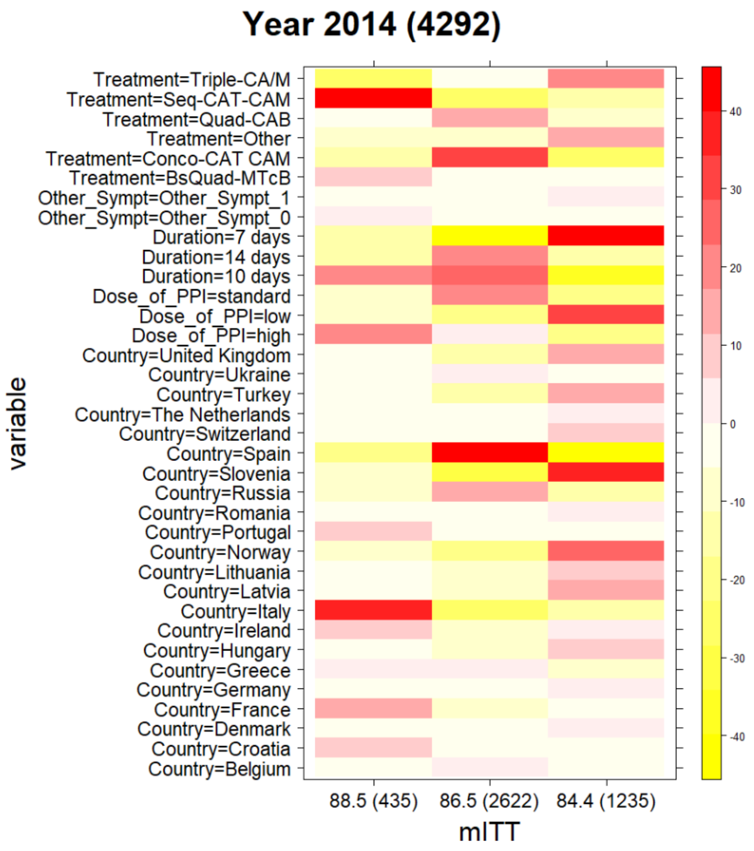

Red colour, high content; yellow colour, low content.

A: amoxicillin; B: bismuth salts; C: clarithromycin; Conco: concomitant; M: metronidazole; Seq, sequential; T: tinidazole; Tc: tetracycline hydrochloride; MTcB was prescribed either in the classical form or as three-in-one single capsule, marketed as Pylera®. The treatment category "other" encompassed (less than 10% of first-line empirical treatments in Europe) and were mainly quadruple therapy with amoxicillin, metronidazole and bismuth (both in Slovenia and Russia) and quadruple therapy with amoxicillin, clarithromycin and josamycin (in Russia only).

Compliance, defined as 1: yes with >90% drug intake or 0: no with <90% drug intake; Dose:of_PPI, defined as low dose PPI: 4.5 to 27 mg OE b.i.d; standard dose PPI: 32 to 40 mg OE b.i.d; high-dose PPI: 54 to 128 mg OE b.i.d; Duration as a duration of treatment of 7, 10 or 14 days; Indication, as ulcer vs dyspepsia; mITT, defined as the modified intention-to-treat; Other_Sympt, defined as other non-frequent gastrointestinal symptoms (0: absence, 1: presence); Sex, as female/male.

In 2014, the highest mITT effectiveness (88.5%) was obtained in cluster #1 (435 cases), mostly with 10-day (99.5%) and high-dose PPIs (59.5%) prescriptions, with sec-CAM/T (85%) therapy, mostly in Italy (64%).

Among the 4,292 patients included in this analysis, significant (p< 0.001) differences were observed between the 3 clusters in the following variables: presence of other gastrointestinal symptoms, duration of treatment, and PPI doses used in prescriptions.

Figure S12. Clusters composition in terms of variables' levels in year 2015.

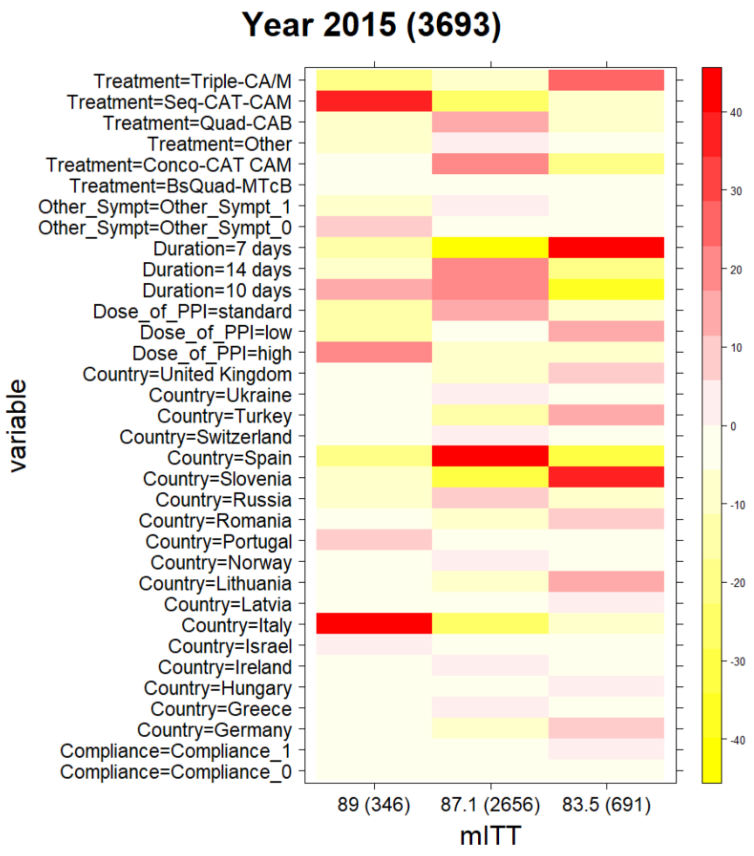

Red colour, high content; yellow colour low content.

A: amoxicillin; B: bismuth salts; C: clarithromycin; Conco: concomitant; M: metronidazole; Seq, sequential; T: tinidazole; Tc: tetracycline hydrochloride; MTcB was prescribed either in the classical form or as three-in-one single capsule, marketed as Pylera®. The treatment category "other" encompassed (less than 10% of first-line empirical treatments in Europe) and were mainly quadruple therapy with amoxicillin, metronidazole and bismuth (both in Slovenia and Russia) and quadruple therapy with amoxicillin, clarithromycin and josamycin (in Russia only).

Compliance, defined as 1: yes with >90% drug intake or 0: no with <90% drug intake; Dose of_PPI, defined as low dose PPI: 4.5 to 27 mg OE b.i.d; standard dose PPI: 32 to 40 mg OE b.i.d; high-dose PPI: 54 to 128 mg OE b.i.d; Duration as a duration of treatment of 7, 10 or 14 days; Indication, as ulcer vs dyspepsia; mITT, defined as the modified intention-to-treat; Other_Sympt, defined as other non-frequent gastrointestinal symptoms (0: absence, 1: presence); Sex, as female/male.

In 2015, the highest mITT effectiveness (88.5%) was obtained in cluster #1 (435 cases), mostly with 10-day (99.5%) and high-dose PPIs (86%) prescriptions, with sec-CAM/T (85%) therapy, mostly in Italy (64%).

Among the 3,693 patients included in this analysis, significant (p< 0.001) differences were observed between the 3 clusters in the following variables: presence of other gastrointestinal symptoms, duration of treatment, and PPI doses used in prescriptions.

Figure S13. Clusters composition in terms of variables' levels in year 2016.

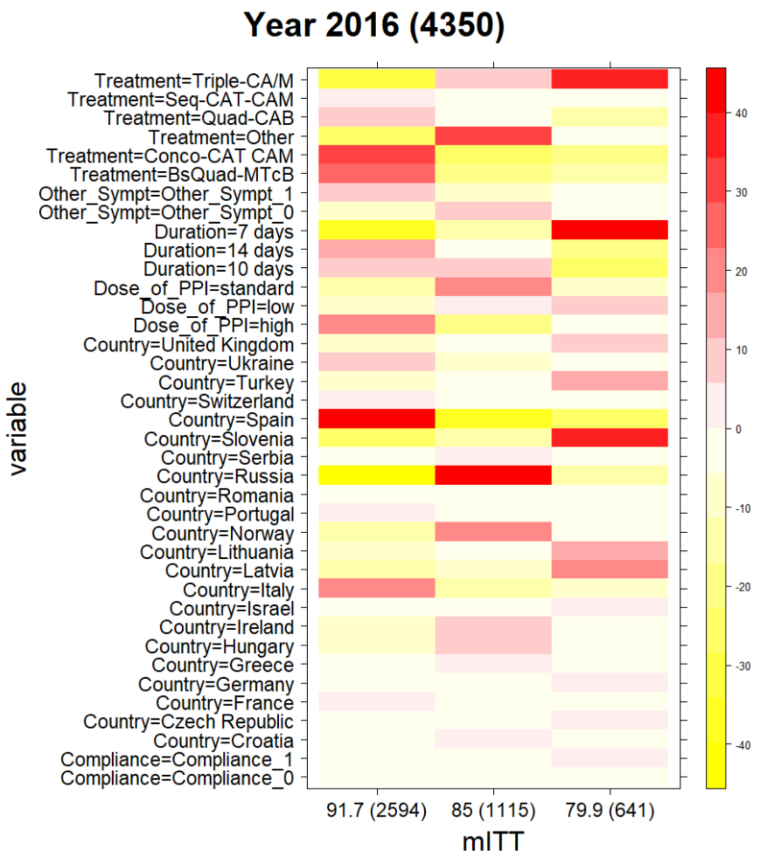

Red colour, high content; yellow colour, low content.

A: amoxicillin; B: bismuth salts; C: clarithromycin; Conco: concomitant; M: metronidazole; Seq, sequential; T: tinidazole; Tc: tetracycline hydrochloride; MTcB was prescribed either in the classical form or as three-in-one single capsule, marketed as Pylera®. The treatment category "other" encompassed (less than 10% of first-line empirical treatments in Europe) and were mainly quadruple therapy with amoxicillin, metronidazole and bismuth (both in Slovenia and Russia) and quadruple therapy with amoxicillin, clarithromycin and josamycin (in Russia only).

Compliance, defined as 1: yes with >90% drug intake or 0: no with <90% drug intake; Dose of_PPI, defined as low dose PPI: 4.5 to 27 mg OE b.i.d; standard dose PPI: 32 to 40 mg OE b.i.d; high-dose PPI: 54 to 128 mg OE b.i.d; Duration as a duration of treatment of 7, 10 or 14 days; Indication, as ulcer vs dyspepsia; mITT, defined as the modified intention-to-treat; Other_Sympt, defined as other non-frequent gastrointestinal symptoms (0: absence, 1: presence); Sex, as female/male.

In 2016, the highest mITT effectiveness (92%) was obtained in cluster #1 (2,523 cases), with 14-day (46%) and low-dose PPIs (34%) prescriptions, with conco-CAM/T (35%) and bismuth quadruple-MTcB (26%) therapies, mostly in Spain (81%).

Among the 4,350 patients included in this analysis, significant (p< 0.001) differences were observed between the 3 clusters in the following variables: presence of other

gastrointestinal symptoms, duration of treatment, PPI doses used in prescriptions and treatment scheme administered.

Figure S14. Clusters composition in terms of variables' levels in year 2017.

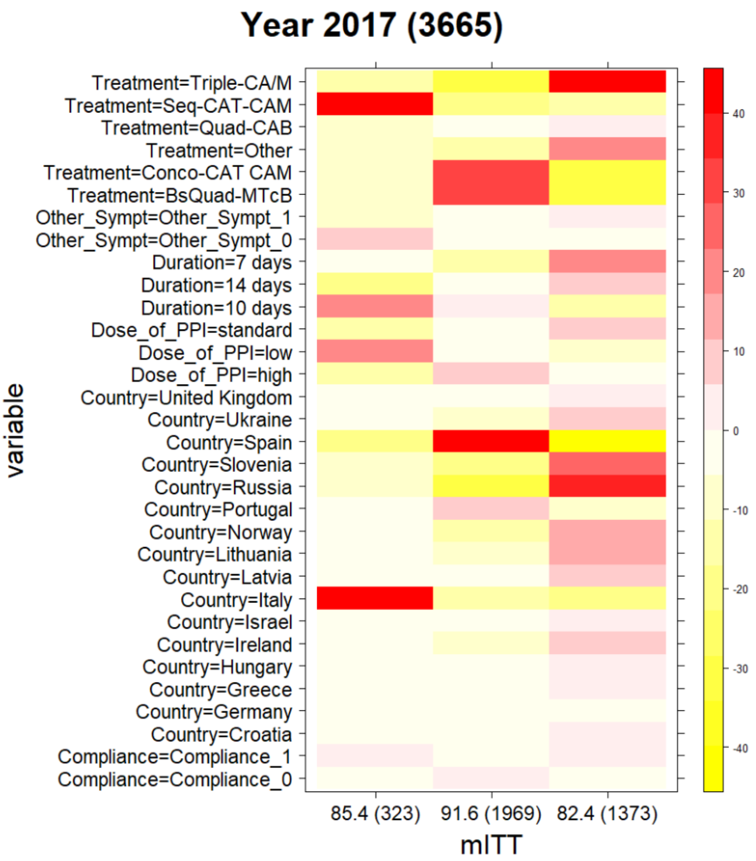

Red colour, high content; yellow colour, low content.

A: amoxicillin; B: bismuth salts; C: clarithromycin; Conco: concomitant; M: metronidazole; Seq, sequential; T: tinidazole; Tc: tetracycline hydrochloride; MTcB was prescribed either in the classical form or as three-in-one single capsule, marketed as Pylera®. The treatment category "other" encompassed (less than 10% of first-line empirical treatments in Europe) and were mainly quadruple therapy with amoxicillin, metronidazole and bismuth (both in Slovenia and Russia) and quadruple therapy with amoxicillin, clarithromycin and josamycin (in Russia only).

Compliance, defined as 1: yes with >90% drug intake or 0: no with <90% drug intake; Dose:of_PPI, defined as low dose PPI: 4.5 to 27 mg OE b.i.d; standard dose PPI: 32 to 40 mg OE b.i.d; high-dose PPI: 54 to 128 mg OE b.i.d; Duration as a duration of treatment of 7, 10 or 14 days; Indication, as ulcer vs dyspepsia; mITT, defined as the modified intention-to-treat; Other_Sympt, defined as other non-frequent gastrointestinal symptoms (0: absence, 1: presence); Sex, as female/male.

In 2017, the highest mITT effectiveness (92%) was obtained in cluster #2 (1,969 cases), with 10-day (58%) and low-dose PPIs (41.5%) prescriptions, with conco-CAM/T (38.5%) and bismuth quadruple-MTcB (43%) therapies, and mostly in Spain (89%).

Among the 3,665 patients included in this analysis, significant (p< 0.001) differences were observed between the 3 clusters in the following variables: presence of other

gastrointestinal symptoms, compliance with treatment, duration of treatment, PPI doses used in prescriptions and treatment scheme administered.

Figure S15. Clusters composition in terms of variables' levels in year 2018.

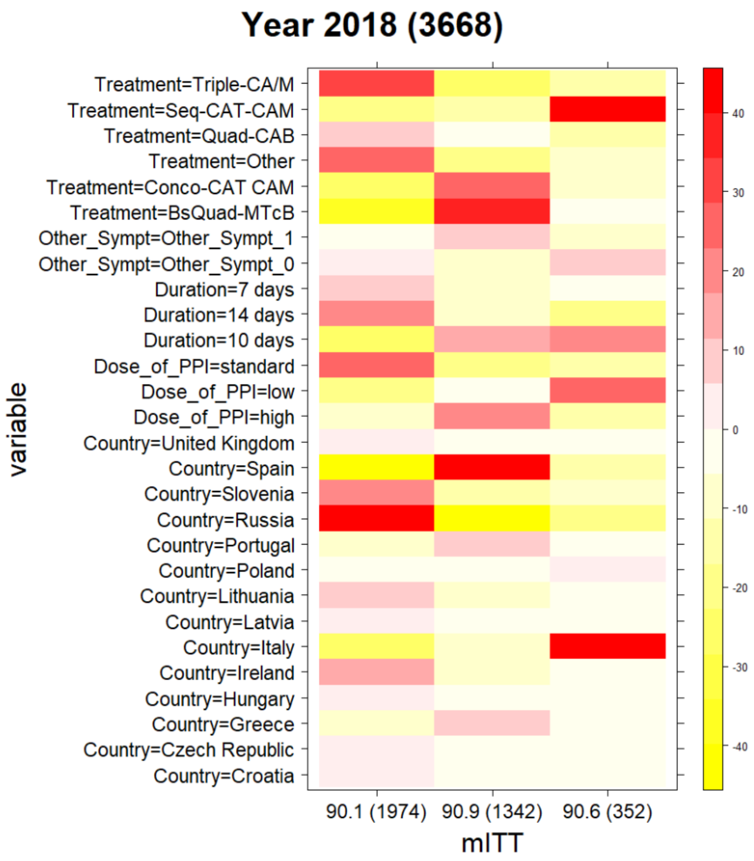

Red colour, high content; yellow colour, low content.

A: amoxicillin; B: bismuth salts; C: clarithromycin; Conco: concomitant; M: metronidazole; Seq, sequential; T: tinidazole; Tc: tetracycline hydrochloride; MTcB was prescribed either in the classical form or as three-in-one single capsule, marketed as Pylera®. The treatment category "other" encompassed (less than 10% of first-line empirical treatments in Europe) and were mainly quadruple therapy with amoxicillin, metronidazole and bismuth (both in Slovenia and Russia) and quadruple therapy with amoxicillin, clarithromycin and josamycin (in Russia only).

Compliance, defined as 1: yes with >90% drug intake or 0: no with <90% drug intake; Dose:of_PPI, defined as low dose PPI: 4.5 to 27 mg OE b.i.d; standard dose PPI: 32 to 40 mg OE b.i.d; high-dose PPI: 54 to 128 mg OE b.i.d; Duration as a duration of treatment of 7, 10 or 14 days; Indication, as ulcer vs dyspepsia; mITT, defined as the modified intention-to-treat; Other_Sympt, defined as other non-frequent gastrointestinal symptoms (0: absence, 1: presence); Sex, as female/male.

In 2018, the highest mITT effectiveness (91%) was obtained in all three clusters, (#1: 1,974 cases, #2: 1,342 cases, #3: 352 cases). Cluster #1 was composed mainly of 14-day (67%) and standard PPI doses (56%) prescriptions, with triple-CA/M therapy (45%) and other non-frequent therapies (33%) mainly in Russia (70%) and Slovenia (14%). Cluster #2 was composed of 10-day (61%) and high-dose PPIs (53%) prescriptions, with bismuth quadruple-MTcB therapy (53%), mostly in Spain (85%). Cluster #3 was

composed of 10-day (98%) and low-dose PPIs (96%) prescriptions, with sec-CAM/T therapy (83%) and mostly in Italy (97% of cases in cluster).

Among the 3,668 patients included in this analysis, significant ($p < 0.001$) differences were observed between the 3 clusters in the following variables: presence of other gastrointestinal symptoms, duration of treatment, PPI doses used in prescriptions and treatment scheme administered.

Figure S16. Clusters composition in terms of variables' levels in year 2019.

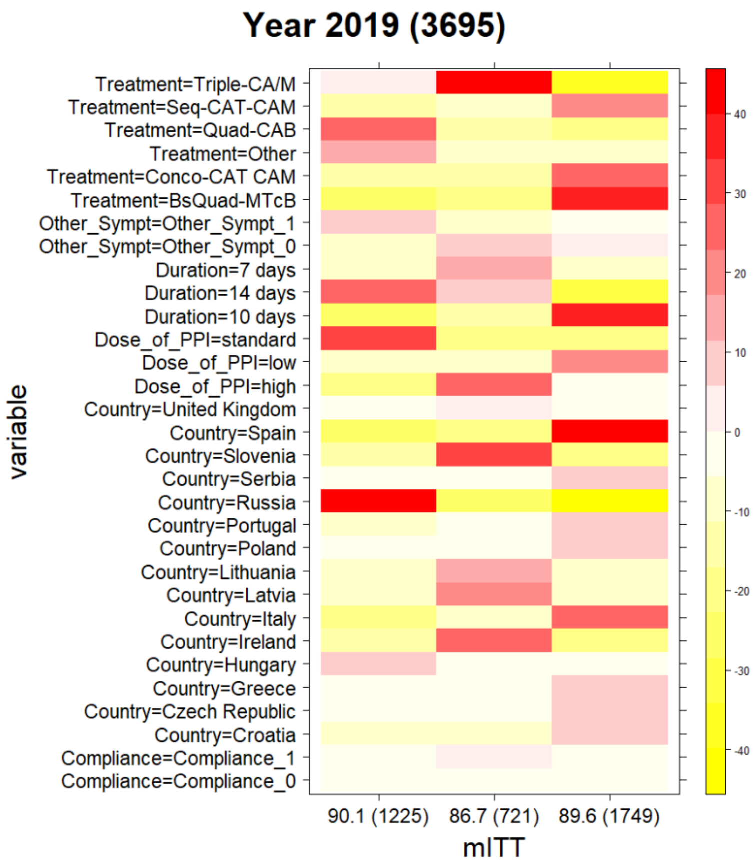

Red colour, high content; yellow colour, low content.

A: amoxicillin; B: bismuth salts; C: clarithromycin; Conco: concomitant; M: metronidazole; Seq, sequential; T: tinidazole; Tc: tetracycline hydrochloride; MTcB was prescribed either in the classical form or as three-in-one single capsule, marketed as Pylera®. The treatment category "other" encompassed (less than 10% of first-line empirical treatments in Europe) and were mainly quadruple therapy with amoxicillin, metronidazole and bismuth (both in Slovenia and Russia) and quadruple therapy with amoxicillin, clarithromycin and josamycin (in Russia only).

Compliance, defined as 1: yes with >90% drug intake or 0: no with <90% drug intake; Dose of_PPI, defined as low dose PPI: 4.5 to 27 mg OE b.i.d; standard dose PPI: 32 to 40 mg OE b.i.d; high-dose PPI: 54 to 128 mg OE b.i.d; Duration as a duration of treatment of 7, 10 or 14 days; Indication, as ulcer vs dyspepsia; mITT, defined as the modified intention-to-treat; Other_Sympt, defined as other non-frequent gastrointestinal symptoms (0: absence, 1: presence); Sex, as female/male.

In 2019, the highest mITT effectiveness (90%) was obtained in cluster #1 (1,225 cases), with 14-day (87%) and standard-dose PPIs (60.5%) prescriptions, with quadruple-CAB therapy (29.5%), mostly in Russia (92%).

Among the 3,695 patients included in this analysis, significant ($p < 0.001$) differences were observed between the 3 clusters in the following variables: presence of other

gastrointestinal symptoms, duration of treatment, PPI dose used in prescriptions and treatment scheme administered.

Figure S17. Clusters composition in terms of variables' levels in year 2020.

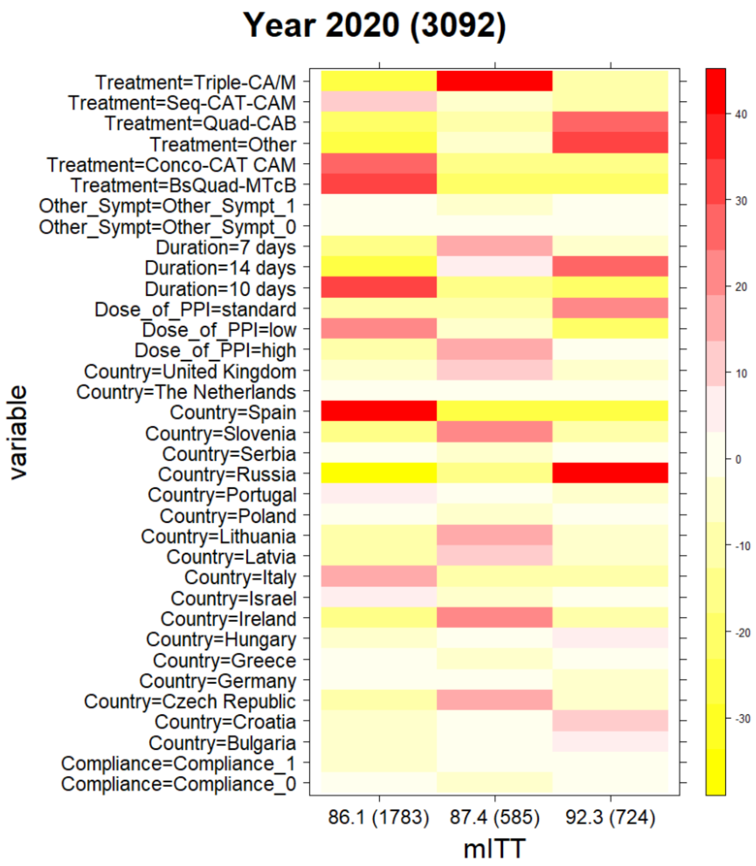

Red colour, high content; yellow colour, low content.

A: amoxicillin; B: bismuth salts; C: clarithromycin; Conco: concomitant; M: metronidazole; Seq, sequential; T: tinidazole; Tc: tetracycline hydrochloride; MTcB was prescribed either in the classical form or as three-in-one single capsule, marketed as Pylera®. The treatment category "other" encompassed (less than 10% of first-line empirical treatments in Europe) and were mainly quadruple therapy with amoxicillin, metronidazole and bismuth (both in Slovenia and Russia) and quadruple therapy with amoxicillin, clarithromycin and josamycin (in Russia only).

Compliance, defined as 1: yes with >90% drug intake or 0: no with <90% drug intake; Dose of_PPI, defined as low dose PPI: 4.5 to 27 mg OE b.i.d; standard dose PPI: 32 to 40 mg OE b.i.d; high-dose PPI: 54 to 128 mg OE b.i.d; Duration as a duration of treatment of 7, 10 or 14 days; Indication, as ulcer vs dyspepsia; mITT, defined as the modified intention-to-treat; Other_Sympt, defined as other non-frequent gastrointestinal symptoms (0: absence, 1: presence); Sex, as female/male.

In 2020, the highest mITT effectiveness (92%) was obtained in cluster #3 (724 cases), with 14-day (88%) and standard PPI doses (57%) prescriptions, with quadruple-CAB therapy (32%) and other non-frequent regimens (61%), and mostly in Russia (82%).

Among the 3,092 patients include4d in this analysis, significant differences were observed between the 3 clusters in the following variables: presence of other

gastrointestinal symptoms ($p<0.05$), compliance with treatment ($p<0.05$), duration of treatment ($p<0.001$), PPI doses used in prescriptions ($p<0.001$) and treatment scheme administered ($p<0.001$).

Figure S18. Clusters composition in terms of variables' levels in year 2021.

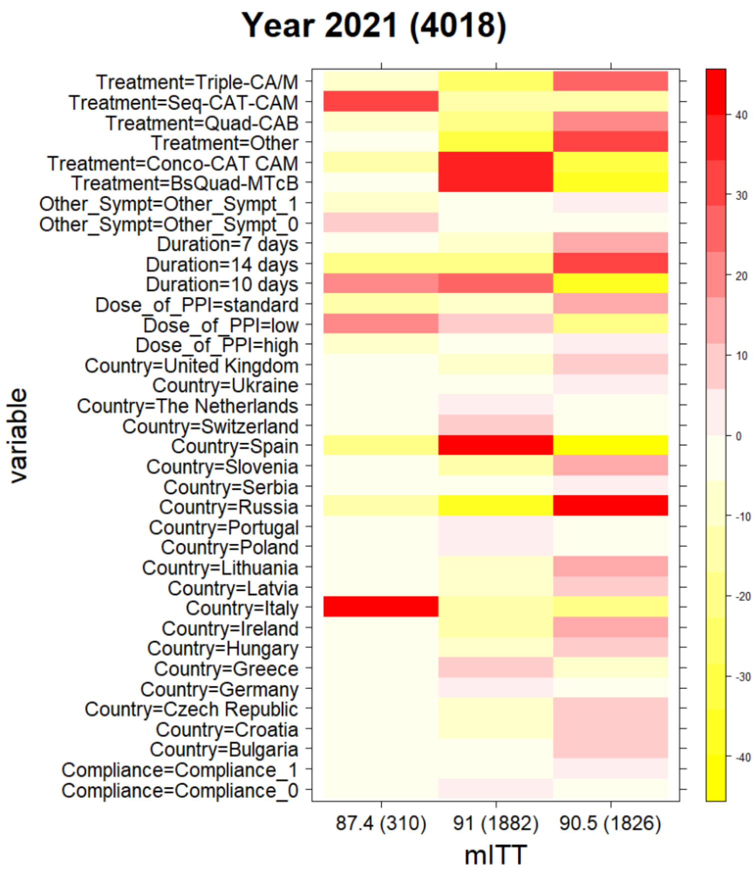

Red colour, high content; yellow colour, low content.

A: amoxicillin; B: bismuth salts; C: clarithromycin; Conco: concomitant; M: metronidazole; Seq, sequential; T: tinidazole; Tc: tetracycline hydrochloride; MTcB was prescribed either in the classical form or as three-in-one single capsule, marketed as Pylera®. The treatment category "other" encompassed (less than 10% of first-line empirical treatments in Europe) and were mainly quadruple therapy with amoxicillin, metronidazole and bismuth (both in Slovenia and Russia) and quadruple therapy with amoxicillin, clarithromycin and josamycin (in Russia only).

Compliance, defined as 1: yes with >90% drug intake or 0: no with <90% drug intake; Dose of_PPI, defined as low dose PPI: 4.5 to 27 mg OE b.i.d; standard dose PPI: 32 to 40 mg OE b.i.d; high-dose PPI: 54 to 128 mg OE b.i.d; Duration as a duration of treatment of 7, 10 or 14 days; Indication, as ulcer vs dyspepsia; mITT, defined as the modified intention-to-treat; Other_Sympt, defined as other non-frequent gastrointestinal symptoms (0: absence, 1: presence); Sex, as female/male.

In 2021, the highest mITT effectiveness (91%) was obtained in cluster #2 (1,882 cases), with 10-day (61.5%) and low-dose PPIs (46%) prescriptions, with bismuth quadruple-MTcB (53%) and with conco-CAM/T (41%) therapies, mostly in Spain (83%).

Among the 4,018 patients included in this analysis, significant differences were observed between the 3 clusters in the following variables: presence of other gastrointestinal

symptoms ($p < 0.001$), compliance with treatment ($p<0.05$), duration of treatment ($p < 0.001$), PPI doses used in prescriptions ($p < 0.001$) and treatment scheme administered ($p < 0.001$).
